# Opioid Signaling in Multiple Sclerosis: Emerging Targets for Repair

**DOI:** 10.3390/ijms27094122

**Published:** 2026-05-05

**Authors:** Renata Perlikowska, Małgorzata Domowicz, Agnieszka Śliwińska, Mariusz Stasiołek

**Affiliations:** 1 Department of Nucleic Acid Biochemistry, Medical University of Lodz, Pomorska 251, 92-213 Lodz, Poland; agnieszka.sliwinska@umed.lodz.pl; 2 Department of Neurology, Medical University of Lodz, Pomorska 251, 92-213 Lodz, Poland; malgorzata.domowicz@umed.lodz.pl (M.D.); mariusz.stasiolek@umed.lodz.pl (M.S.)

**Keywords:** multiple sclerosis, opioid signaling, immunomodulation, remyelination, neuroprotection

## Abstract

Multiple sclerosis (MS) is a chronic immune-mediated disorder of the central nervous system (CNS) characterized by persistent inflammation, demyelination, and progressive neurodegeneration, driven largely by aberrant activation of T and B lymphocytes that infiltrate the CNS and cause myelin and axonal damage, leading to neurological impairment. Although current therapies broadly suppress immune activity and reduce relapse rates, their effects on neurodegenerative processes remain limited. Also, the safety profile of disease-modifying therapies (DMTs) may become problematic, especially in older patients with comorbidities and/or advanced disability. Emerging data suggest that opioid signaling may exert immunomodulatory, remyelinating, and neuroprotective effects, representing a novel and underexplored therapeutic avenue. Given that current MS therapies primarily target inflammation but fail to promote myelin repair or prevent neurodegeneration, opioid signaling emerges as a novel and underexplored pathway with potential benefits for immunomodulation and remyelination, as well as possible neuroprotective effects. Despite concerns about classical opioid-related adverse effects, accumulating evidence shows that opioid-mediated interventions have been associated with reduced inflammatory activity, attenuation of demyelination, and enhanced neuronal survival and have shown therapeutic benefit in MS. Although current findings are largely preclinical, they provide a compelling rationale for further investigation of the opioid system as a potential adjunctive or novel therapeutic strategy.

## 1. Introduction

Multiple sclerosis (MS) is a chronic, immune-mediated disease of the central nervous system (CNS) characterized by inflammation, demyelination, and neurodegeneration [1]. Its epidemiology has significantly transformed over recent years. The disease affects an estimated 2.8 to 2.9 million people globally, and its prevalence continues to rise due to advances in diagnostic techniques, increased life expectancy, greater disease awareness, and longer survival among patients [2,3,4]. Simultaneously, the MS population has been aging, with peak prevalence observed in individuals over 50–60 years of age. Typically, the disease presents between the ages of 20 and 40; it is one of the most common neurological disorders in young adults and a leading non-traumatic cause of long-term disability in this age group [2]. A clear gender disparity is evident, with women affected approximately three times more often than men, particularly in relapsing-remitting MS (RRMS) [5,6]. The marked female predominance in MS likely reflects the interplay of sex hormones, particularly estrogen- and progesterone-driven immune modulation, and intrinsic sex-related differences in immune responsiveness driven by X-linked immune genes and the hormonal sensitivity of immune cells [7]. Geographically, MS is more prevalent in Northern and Western Europe, North America, and other regions farther from the equator, where environmental factors may contribute to disease risk. The latitude effect, linked to vitamin D status, is a robust early-life environmental risk factor for MS [8].

**Figure 1:**
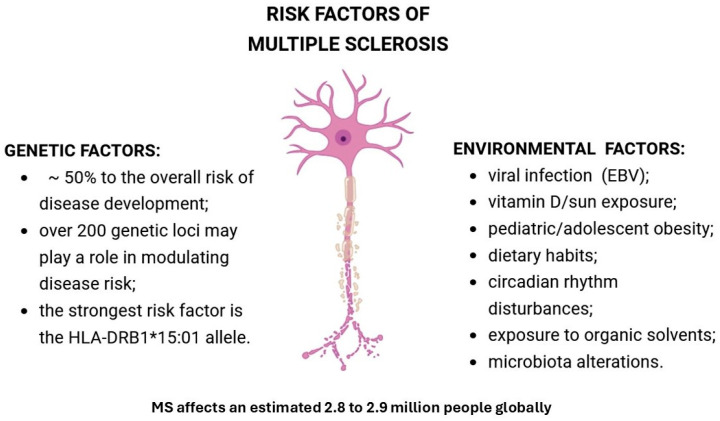
Risk factors for multiple sclerosis (MS). Created in https://BioRender.com.

Although the etiology of MS remains incompletely understood, genetic factors are estimated to account for approximately 50% of disease risk [9], with the strongest associations within the major histocompatibility complex (MHC) class II region on chromosome 6, particularly the human leukocyte antigen (HLA) HLA-DRB1*15:01 allele [10]. This allele influences antigen presentation to CD4^+^ T cells and promotes autoimmune responses against myelin, conferring an approximately threefold increased risk of MS [10]. Familial cases account for about 12.6% of MS [11], with risk increasing with genetic relatedness and potentially greater disease severity compared to sporadic MS [9]. In addition, other immune-regulatory genes, including IL7RA and IL2RA, have been associated with MS susceptibility [12]. Current estimates suggest that over 200 genetic loci may play a role in modulating disease risk [13]. Genetic and environmental risk factors in MS are summarized in Figure 1.

Alongside genetic predisposition, environmental factors are also recognized as important contributors to disease onset and progression [10]. Strong evidence shows that viral infection, particularly Epstein–Barr virus (EBV), is necessary for MS development, especially in genetically susceptible individuals [14,15], while EBV infection and HLA-DR15 act synergistically to increase MS risk by impairing immune tolerance to myelin [16]. Low vitamin D levels are associated with increased MS risk and activity, whereas supplementation is associated with reduced relapse rates in RRMS, likely due to immunomodulatory effects [14,15,16,17,18]. Additional environmental risk factors include smoking and oral tobacco use [19], as well as childhood and adolescent obesity [20,21]. Dietary habits also contribute to disease modulation, particularly poor dietary practices such as high saturated fat intake, low fiber and calcium consumption, and low adherence to the Mediterranean diet [22]. Diets high in saturated fats may promote autoimmunity, whereas omega-3 fatty acids appear to exert protective effects. Inflammation and metabolic disorders may also result from circadian rhythm disturbances (e.g., shift work) [23]. Additional contributors include exposure to organic solvents [24] and gut microbiota alterations [25], both of which may influence immune homeostasis and neuroimmune interactions. Interestingly, research data emphasize a gene–environment interaction, where genetic predisposition (e.g., HLA-DRB1*15:01) is modulated by environmental exposures like EBV [16] and vitamin D levels [26].

**Figure 2:**
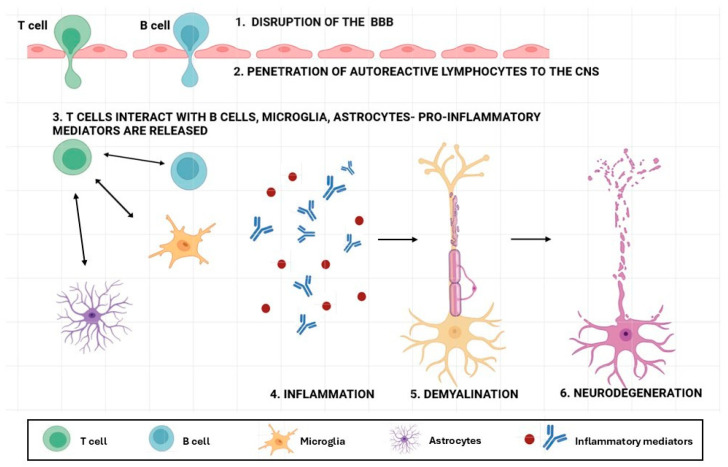
Key stages of MS pathogenesis: blood–brain barrier (BBB) disruption (1), lymphocyte entry into the central nervous system (CNS) (2), inflammatory cascade (3), inflammation (4), demyelination (5), and neurodegeneration (6). Created in https://BioRender.com.

In MS, immune tolerance to myelin components is lost, leading to an autoimmune response that initiates CNS damage. This process is amplified by activated glial cells, particularly microglia and astrocytes, which release pro-inflammatory mediators and promote the recruitment of peripheral immune cells, including T and B lymphocytes, into the CNS (Figure 2). The resulting inflammatory cascade leads to demyelination and axonal injury and accelerates neurodegeneration. Over time, inflammation becomes increasingly compartmentalized within the CNS and is driven by chronic glial activation and intrinsic CNS factors, contributing to sustained functional decline and underscoring the need for therapies that address immune dysregulation, myelin repair, and neuroprotection.

Clinically, MS is classified into RRMS and progressive forms of the disease, including primary MS (PPMS) and secondary progressive MS (SPMS). Although progression ultimately affects all patients, it may remain subclinical early in the disease course and become more apparent with increasing age and disease duration. MS presents with diverse symptoms, including motor and sensory deficits, visual disturbances, impaired coordination, bladder and bowel dysfunction, chronic pain, cognitive impairment, and mood disorders.

Due to the lack of fully specific symptoms and biomarkers, MS diagnosis relies on clinical presentation supported by MRI, cerebrospinal fluid (CSF) analysis, and exclusion of alternative diagnoses [27,28,29,30,31]. Successive revisions of the McDonald criteria aimed to enable earlier diagnosis without compromising accuracy. The 2024 criteria allow MS diagnosis in asymptomatic individuals based on typical MRI findings combined with additional features, such as positive CSF results, the Central Vein Sign (CVS), or evidence of “dissemination in time” (DIT) [32]. Notably, the optic nerve was introduced as a fifth anatomical region for assessing “dissemination in space” (DIS), and the definitions of both DIS and DIT were revised. Intrathecal immunoglobulin synthesis remains the most informative laboratory marker and is traditionally assessed by CSF-specific oligoclonal bands (OCBs), with κ-free light chains (κ-FLCs) now accepted as an equivalent alternative. Among other biomarkers, neurofilament light chains (NfLs) reflect acute axonal injury and inflammatory activity [33], whereas glial fibrillary acidic protein (GFAP) indicates astrocytic activation and is associated with progressive disease and disability [34]. Chitinase-3-like protein 1 (CHI3L1) is linked to chronic smoldering inflammation and disease progression [35], while autoantibodies such as anti-myelin oligodendrocyte glycoprotein (MOG)-Ab and anti-aquaporin-4 (AQP4) serve as differential diagnostic markers to distinguish MS from MOG-associated disease and neuromyelitis optica spectrum disorder [36,37].

Accurate diagnosis is essential for selecting therapies that limit inflammatory activity and slow neurodegeneration in MS. Early initiation of DMTs is associated with reduced relapse rates and delayed disease progression. Current recommendations emphasize early intervention, individualized therapy selection, and ongoing monitoring to balance treatment efficacy and safety [29,30,31]. Advances in DMTs over recent decades have reshaped therapeutic strategies, with studies highlighting the importance of high-efficacy treatments (HETs) in the earliest stages of MS as a key factor in slowing disease progression [38].

However, there are still no therapies effectively preventing disability accumulation in all patients, and the efficacy of most disease-modifying treatments (DMTs) varies with increasing patient age and disease duration. Additionally, due to the immunosuppressive mechanisms of action of available DMTs, safety concerns, including risks of infection and malignancy, arise, which are particularly relevant during long-term treatment of older patients and those with comorbidities and/or significant disability [39,40].

The search for new therapies is therefore ongoing, and emerging evidence shows that the endogenous opioid system plays a multifaceted role in modulating immune responses, neuroinflammation, and neuronal survival, all of which are central to the pathophysiology of MS, thereby opening new therapeutic avenues. Traditionally recognized for its role in pain regulation, opioid signaling is now understood to exert far broader physiological effects, including the modulation of reward and motivation, stress responsiveness, immune and inflammatory processes, gastrointestinal function, and endocrine/hormonal regulation, while also playing an increasingly recognized role in controlling glial cell activity, cytokine production, and BBB integrity [41]. Opioid receptors, including μ-opioid (MOR), δ-opioid (DOR), κ-opioid (KOR), and nociceptin/orphanin FQ (NOP) receptors, may be expressed constitutively or induced, depending on the specific cell type (neuronal or non-neuronal) and physiological conditions, positioning them as potential modulators of both peripheral and central immune responses. In MS, where chronic inflammation, demyelination, and progressive neurodegeneration coexist, the ability of opioid receptors and their ligands to influence immune cell behavior, remyelination pathways, and neuroprotection presents a compelling therapeutic opportunity. Current evidence shows that KOR activation is associated with the promotion of myelin repair and attenuation of microglial activation [42], while DOR signaling may attenuate oxidative stress and promote neuronal resilience [43]. Moreover, strategies that enhance endogenous opioid activity, such as the use of low-dose naltrexone (LDN), have shown preliminary promise in improving quality of life and reducing symptom burden in patients with MS [44,45].

Given the limitations of current MS therapies, which largely target inflammation but fail to sufficiently halt neurodegeneration or promote repair, opioid signaling represents a novel and underexplored area with potential dual immunomodulatory and remyelination-promoting effects, as well as possible neuroprotective benefits. The medical use of opioid receptor ligands is limited by concerns about classical adverse effects such as tolerance and addiction. However, to highlight their therapeutic potential, which may offer benefits that outweigh their associated risks, this review provides a comprehensive characterization of the opioid system and evaluates growing evidence that endogenous and exogenous opioid receptor ligands can exert significant immunomodulatory, remyelination-enhancing, and neuroprotective activity in MS. A deeper understanding of this system could pave the way for innovative therapeutic approaches that complement existing treatments and address unmet clinical needs in MS.

## 2. From Pathophysiology to Therapy

MS is a complex autoimmune and neurodegenerative disease that results from dysregulated communication between the CNS and the immune system and an abnormal immune response. Despite extensive research efforts, the identity of the initiating autoantigen and the mechanisms driving neuro-immune dysregulation remain unresolved. However, a central event in MS pathogenesis is the disruption of the BBB, which permits autoreactive T and B lymphocytes to penetrate the CNS, where their interactions with antigen-presenting cells trigger the release of pro-inflammatory and cytotoxic mediators. The penetration of immune cells into the CNS becomes possible through the destruction of the BBB, mediated by matrix metalloproteinase (MMP-2 or MMP-9) activation, increased adhesion molecules, and the loss of tight-junction proteins. T cells are key drivers of MS pathology, together with Th17 (CD4^+^) cells, producing cytokines such as IL-17, IL-6, and IL-22, and representing a dominant pathogenic subset that promotes extensive inflammatory cell recruitment and further BBB disruption [46]. Additional contributors include Th1 (CD4^+^) cells, which secrete IFN-γ and TNF-β and activate macrophages, leading to oligodendrocytes and myelin damage, as well as CD8^+^ T cells, which produce IL-17 and exert direct cytotoxic effects on oligodendrocytes and neurons. Moreover, B lymphocytes are also involved in the pathogenesis of MS through antigen presentation, the production of proinflammatory cytokines (IL-6 or TNF-α), and the formation of B lymphocyte aggregates in the meninges, which drive cortical demyelination and neuronal loss. Their crucial role is also emphasized by the high clinical efficacy of B lymphocyte depletion therapies, i.e., monoclonal antibodies against the CD20 antigen [47]. The processes are accompanied by a reduced concentration of regulatory cytokines, such as IL-10 and TGF-β, which mirrors the pathological immune dysregulation in MS, including diminished immune tolerance and persistent pro-inflammatory activity, contributing to chronic neuroinflammation and demyelination.

Once inflammation is initiated, CNS-resident cells become activated. Triggered microglia continuously generate inflammatory mediators and reactive oxygen species (ROS), while responsive astrocytes amplify inflammation, form glial scars, and impede regenerative processes. These events drive tissue-level damage, which is characterized by demyelination, axonal transection, neurodegeneration, reactive astrocytosis, and meningeal inflammation, affecting both white and gray matter [48].

Histopathologically, MS manifests as active plaques with dense inflammatory infiltrates and ongoing myelin destruction, as well as chronic active (smoldering) plaques with a rim of activated microglia [49]. Furthermore, there are also inactive plaques, marked by gliotic, hypocellular demyelinated tissue with significant axonal loss, and partially repaired shadow plaques, reflecting incomplete remyelination. Thus, MS is characterized by a complex interplay of molecular, cellular, and tissue-level abnormalities that culminate in characteristic pathomorphological lesions, as summarized in Table 1. Together, these changes illustrate the multifocal, immunological, demyelinating, and neurodegenerative nature of MS.

Characterizing molecular, cellular, and tissue-level alterations in MS is essential for identifying the mechanisms driving disease progression and therapeutic targets. Emerging treatments may address diverse processes, including BBB dysfunction and systemic factors such as vitamin D deficiency and microbiome dysregulation. However, in this review, we focus on three key clinical objectives for novel MS treatments: (1) suppressing pathological immune activation, (2) promoting remyelination, and (3) preventing neurodegeneration.

In general, current and emerging therapies for MS aim to reduce immune activation and CNS infiltration by autoreactive T and B lymphocytes. Mechanisms such as antiproliferative effects, functional sequestration of lymphocytes within lymph nodes, inhibition of lymphocyte migration across the BBB, depletion of pathogenic B cells, immune reconstitution, and modulation of inflammatory signaling pathways are used [50,51]. Such control of neuroinflammation limits the formation of new inflammatory lesions and decreases relapse frequency. In turn, remyelination may be achieved by promoting the differentiation and maturation of oligodendrocyte precursor cells (OPCs), thereby enabling the repair of damaged myelin sheaths [50,51]. Effective remyelination restores conduction velocity and protects axons from further degeneration. Targeted approaches include the removal of molecular inhibitors of OPC differentiation, modulation of developmental signaling pathways, and promotion of lipid and myelin synthesis. These therapies could, to some degree, reverse existing disability and modify the long-term course of MS to a greater extent than immunosuppressive therapy alone [52]. Since early neurodegenerative processes are recognized in MS, protection of axons, neurons, and oligodendrocytes is expected to reverse or at least slow disease-related injury. Neuroprotective strategies focus on stabilizing mitochondrial function, reducing excitotoxicity, modulating microglial activation, and preventing oxidative stress. Achieving neuroprotection is essential for preserving long-term functional capacity, especially in progressive forms of MS, in which inflammation is less prominent, and current immunomodulatory therapies provide limited benefit [50,53].

Recognizing the complex interactions among CNS inflammation, immune dysregulation, demyelination, and neurodegeneration in MS, we propose that the opioid system, expressed in neuronal, glial, and immune cells, and acting as a bidirectional bridge between the CNS and the immune system, might be a topic worth exploring. Evidence shows that opioid ligands and receptors modulate neuroinflammation, immune activation, and chronic pain. Dysregulation of opioidergic signaling may contribute to disease pathogenesis, whereas specific pathways, such as KOR activation, demonstrate promising promyelinating and neuroimmunomodulatory effects [41,42]. Collectively, these findings position the opioid system as a mechanistic and potentially therapeutic link between CNS pathology, immune dysfunction, and the clinical manifestations of MS.

## 3. Endogenous Opioid Signaling

**Figure 3:**
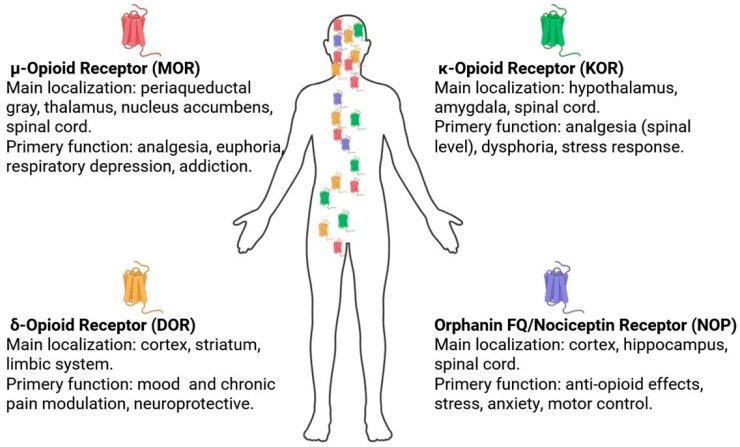
Locations of the opioid receptors. Created in https://BioRender.com.

The endogenous opioid system is a crucial component of the body’s neurochemical network that helps regulate a wide range of physiological and psychological processes. It is integral to the regulation of nociception, mood, emotion, and motivational states; it also modulates the stress response, neuroendocrine activity, appetite and eating behavior, as well as immune and cognitive functions [54]. The opioid system acts mainly symptomatically, causing changes in physiological responses and signaling without causally modifying disease-related alterations. Opioid receptors, MOR, DOR, KOR, and NOP, can be expressed both constitutively, through basal promoter activity, and in an inducible manner, involving transcriptional regulation through signaling pathways (e.g., nuclear factor kappa-light-chain-enhancer of activated B cells (NF-κB) or mitogen-activated protein kinase (MAPK)) triggered by inflammatory mediators. They are broadly distributed across the central and peripheral nervous systems under normal conditions [55]. Figure 3 presents a map of the localization of opioid receptors in the nervous and peripheral systems, along with a brief description of their main functions.

The opioid system is traditionally recognized for its role in pain modulation; thus, in neurons, opioid receptors, especially MOR, are densely distributed in pain-control regions, such as the periaqueductal gray, thalamus, and cortex [Figure 3], where their activation inhibits nociceptive transmission and mediates analgesia [56,57]. Additionally, opioid receptors are expressed in brain areas involved in mood regulation (cortex, amygdala, hippocampus, nucleus accumbens, hypothalamus), contributing to affective processing and emotional states. They are predominantly localized on presynaptic terminals, where they modulate vesicular neurotransmitter release, and on postsynaptic membranes, influencing excitability and synaptic plasticity via G-protein-mediated ion-channel modulation [58]. Overall, opioid receptors are present in multiple brain regions, including the cortex, thalamus, and spinal cord, as well as within functional circuits such as cortical, limbic, and midbrain networks [59,60].

Opioid receptors have also been identified in non-neuronal cells of the nervous system, known as glial cells (microglia and astrocytes), which support both the central and peripheral nervous systems and play crucial roles in immune-like functions [61]. Their expression levels vary depending on glial cell type, brain region, species, and conditions such as chronic opioid exposure or neuroinflammation [62]. Current knowledge regarding the involvement of microglia and astrocytes largely derives from studies employing models of chronic opioid exposure [63,64,65,66]; thus, these models may not fully reflect physiological conditions, as chronic opioid exposure can induce adaptive changes in glial cells that often lead to overexpression and upregulation of opioid receptors, actively promoting neuroinflammation. Therefore, the basal level expression levels and functions of opioid receptors in this context may potentially confound interpretation.

Interestingly, opioid receptors are not limited to neurons and glial cells. Beyond their roles in pain, mood, and addiction regulation, they modulate immune dynamics and influence neuroinflammatory and neuroimmune processes, highlighting their broad physiological roles [67,68]. In the immune system, the presence of opioid receptors has been demonstrated at both the mRNA and protein levels and supported by functional assays [61,69,70,71]. Among immune cells, those expressing opioid receptors include B [72,73] and T lymphocytes [74,75], macrophages [61,71], and microglia [66,76], as mentioned above. Although opioid receptors play a modulatory role in immune function, they are constitutively expressed at low levels in lymphoid cells. The paper by Zhang et al. [77] supported the idea that MOR expression is relatively low in developing T cells but can be induced under specific pathological conditions (via anti-CD3/CD28 co-stimulation or exposure to cytokines like interferon gamma (IFN-γ), interleukin-1 beta (IL-1β), interleukin-2 (IL-2), transforming growth factor beta (TGF-β), suggesting that receptor regulation is influenced by the immune microenvironment. Generally, opioid receptor expression can be increased in immune cells, glia, and even peripheral sensory neurons during inflammation or tissue damage [61]. Therefore, since the opioid system responds to inflammation, its investigation as a potential target to slow or halt the progression of MS may be justified; however, additional mechanistic and functional evidence is needed. Stress or chronic exposure to opioids can also alter receptor density and distribution [78,79]. Moreover, immune and endothelial cells are susceptible to induction of opioid receptor expression under the influence of cytokines and growth factors [71,80]. Broadly, evidence regarding opioid receptor expression across different cell types remains inconclusive and controversial, highlighting the need for further well-controlled studies to verify the presence of all receptor subtypes.

Endogenous opioid peptides are synthesized and released by multiple cell types, contributing to diverse physiological and immunological functions. Neurons represent the primary source of opioid peptides within the central and peripheral nervous systems, releasing, among others, β-endorphin, enkephalins, and dynorphins, typically in response to depolarization and calcium influx [81]. These peptides modulate nociceptive transmission, stress responses, and neuroprotective pathways. Glial cells release primarily enkephalins and dynorphins in response to injury or neuroinflammation, influencing synaptic plasticity and neuroimmune interactions [82,83]. Immune cells produce and secrete enkephalins and endorphins during inflammatory or immune activation, providing local analgesia and exerting immunomodulatory effects through cytokine regulation [61,72,84]. Collectively, the opioid system operates as a dynamic network across neuronal, immune, and glial compartments, integrating pain modulation with immune and neuroprotective functions.

Typically, once activated by an endogenous or exogenous ligand, the opioid receptor undergoes conformational changes and consequently initiates several downstream effects, including inhibition of adenylyl cyclase (AC) activity, leading to reduced cyclic AMP (cAMP) levels, and subsequent reduction of downstream protein kinase A (PKA) activity [85]. Additionally, opioid receptor activation regulates calcium and potassium ion channels, leading to decreased neuronal excitability and reduced neurotransmitter release [57]. Moreover, changes in cAMP/PKA signaling and related downstream pathways can modify transcription factors such as cAMP response element-binding protein (CREB), resulting in altered gene expression and long-term adaptations in neurons and glial cells. Depending on the receptor subtype and location, these effects include analgesia (especially via MOR), euphoria (MOR in the reward pathway), sedation, respiratory depression, reduced gastrointestinal motility, as well as hormonal and immune modulation.

In addition to classical opioid receptors, non-classical receptors such as the opioid growth factor receptor (OGFr) have been identified in immune and neural cells, mediating distinct biological functions beyond analgesia [86,87]. The receptor is primarily located on the outer nuclear membrane and within the nucleoplasm, unlike classical opioid receptors, which are membrane-bound; however, this positioning allows OGFr to directly influence cell-cycle regulation by interacting with transcriptional machinery and cyclin-dependent kinase inhibitors [88]. OGFr interacts with opioid growth factor (OGF, also known as Met-enkephalin) to regulate cell proliferation and tissue homeostasis, playing a role in processes such as wound healing and cancer biology. Furthermore, opioid-mediated immunomodulation involves complex mechanisms, including modulation of cytokine release, alteration of immune cell activity, and regulation of inflammatory pathways through both central and peripheral opioid receptors. These effects highlight the broader physiological significance of the opioid system, extending from neuroregulation to immune function and, as discussed later in this review, serving as a foundation for exploring novel therapeutic strategies for MS.

## 4. Opioid Signaling and the Immune System

Given the potential application of opioid signaling in MS therapy, understanding the complex interactions between the opioid system and immune mechanisms becomes crucial. Opioid signaling exerts a complex and multifaceted influence on the immune system, modulating both immune responses and inflammatory processes, with both immunosuppressive and immunostimulatory potential [41,72]. In immune cells, opioid receptors display unique expression and functional patterns. They are typically absent in resting cells or present at very low basal levels (e.g., KOR) [89], but can be upregulated upon activation, such as through CD3/CD28 co-stimulation or exposure to cytokines (IFN-γ, IL-1β, IL-2, IL-4), or growth factors (tumor necrosis factor (TNF), TGF-β) (e.g., MOR) [61,71,90,91]. Once induced, these receptors traffic to the plasma membrane and undergo internalization following ligand binding. Activation of MOR in human T cells significantly increases intracellular cAMP, which activates PKA, ultimately inhibiting lymphocyte-specific protein tyrosine kinase (Lck) and early T-cell receptor (TCR) signaling. This leads to reduced IL-2 transcription, inhibition of activator protein-1 (AP-1), nuclear factor of activated T cells (NFAT), NF-κB activation, and overall immunosuppression [90,91]. The signaling is also associated with inhibition of calcium flux, MAPK activation, and phosphorylation of key signaling proteins. DOR and KOR also modulate immune responses through mechanisms likely involving Gα_i_/_o_ signaling, although these pathways are less clearly defined [68,92].

Immune cells express opioid receptors and can both produce and respond to opioids. They are capable of stimulating the release of, or enhancing the synthesis of, endogenous opioid peptides, which may bind to opioid receptors on peripheral sensory neurons, contributing to local analgesia. Overall, different opioids can exert varied effects; some are immunosuppressive, others immunostimulatory, and some demonstrate dual effects [93]. They can impair functions of macrophages, natural killer cells, and T cells [72], interact with cytokine production and immune cell signaling [94], and potentially increase vulnerability to infections through suppression of immune responses [95]. However, more controlled clinical studies are needed to fully elucidate these complex interactions.

When considering the spectrum of opioids that can be produced within the immune system, it is important to highlight that macrophages, monocytes, granulocytes, and both T and B lymphocytes have been shown to express mRNA for β-endorphin, its precursor pro-opiomelanocortin, and proenkephalin [96,97]. In peripheral inflamed tissue, leukocytes are the main and best-studied source of opioid peptides, where inflammatory mediators such as IL-1β and corticotropin-releasing factor (CRF) stimulate the release of β-endorphin and enkephalins, which act on peripheral neurons to produce analgesia [98,99]. These findings show that inflammation can paradoxically activate endogenous pain-relief pathways, leading to peripheral antinociception, while simultaneously suppressing neuroinflammation and avoiding the liabilities associated with central opioid mechanisms. In contrast, opioid-mediated modulation of pain and inflammation in the CNS follows a different pathway, relying primarily on neurons and glial cells rather than leukocytes as the principal opioid-releasing populations. Furthermore, opioid peptide release is also observed following noradrenaline stimulation, linking catecholaminergic signaling with endogenous analgesic activity and showing that central pain-relief mechanisms are integrated with the body’s stress and arousal systems [99,100,101].

Opioids modulate immune function through direct and indirect mechanisms. In 1979, the direct effects of opioids on the immune system were described [102], with morphine generally exerting immunosuppressive effects, whereas Met-enkephalin appears to enhance or modulate immune activity. Notably, both effects on T cells were reversed by naloxone, confirming the involvement of opioid receptors. Subsequent studies in 1988 demonstrated the presence of opioid receptors on the surface of immunocompetent cells [103]. These discoveries became the starting point for later research, which ultimately yielded much more evidence for the presence and role of all four classical opioid receptor subtypes in various types of immune cells, including both animal and human immune cell lines, as well as immune cells isolated from untreated animals and healthy human donors [41,69,70,73,95]. So far, direct interactions with opioid receptors expressed on immune cells have been confirmed [68,71,72,104,105]. MOR has the strongest evidence base, with specificity confirmed through in vitro cell models [106,107], in vivo MOR knockout mice that fail to demonstrate immunosuppressive responses to morphine, and highly selective MOR agonist peptides like [D-Ala^2^,N-Me-Phe^4^,Gly^5^-ol]-enkephalin (DAMGO) [107,108]. Leukocytes within inflamed tissues serve as an example of cells that secrete opioid peptides in response to ongoing inflammation and pathological pain, providing local analgesia by activating opioid receptors on peripheral nociceptors [96,109,110]. This mechanism illustrates a direct neuroimmune interaction, as immune-derived opioids not only modulate inflammatory pain but also alleviate neuropathy-induced mechanical allodynia in mice by acting on nociceptor-expressed receptors at sites of nerve injury [111].

Immunomodulation also occurs through indirect mechanisms related to neuroendocrine regulation, though the evidence is mixed and complex. The first reports on this topic suggested that chronic opioid exposure alters immune function primarily through activation of the hypothalamic–pituitary–adrenal (HPA) axis and the sympathetic nervous system, thereby modulating cortisol levels and stress responses [112,113]. This view was challenged by M. Al-Hashimi et al. [105], who argued that the evidence for HPA axis activation is weak and varies across species. However, N. C. Alonzo et al. [114] emphasized that the interaction between the CNS, the autonomic nervous system, and the HPA axis is crucial to understanding the differential effects of opioids on immunity. This mechanism appears to involve central opioid receptors, which indirectly influence immune parameters, but I. Welters et al. [115] noted that existing clinical and experimental data remain preliminary and inconclusive; therefore, further research is needed to definitively characterize the HPA axis-mediated immunomodulatory mechanisms of opioids.

## 5. The Crosstalk Between Opioid Signaling and the Immune System

Opioid signaling demonstrates robust functional crosstalk with the immune system through diverse molecular and cellular mechanisms. Numerous studies have explored how activation of opioid receptors influences immune signaling pathways, providing insight into how opioid systems modulate immune responses beyond their classical role in analgesia [116,117]. This neuroimmune crosstalk is mediated by conserved opioid peptides and receptors, which serve as key messengers between the nervous and immune systems.

Research indicates a role for MOR engagement in modulating immune activation, and this effect has been assessed not only in general immune contexts but also in chronic diseases such as HIV. It has been established that individuals with HIV undergoing methadone treatment exhibit significantly elevated markers of immune activation and inflammation compared to those not receiving methadone, implicating MOR activation in the persistence of immune stimulation in these patients [118]. This sustained immune activity may contribute to disease progression, linking opioid use to intensified inflammatory responses [119]. McCarthy et al. [104] found that opioids alter both innate and acquired immune responses, affecting phagocytic activity and cytokine expression. Accumulated evidence shows that activation of opioid receptors can alter resistance to HIV, suggesting a potential direct impact on HIV infection. Opioids have been shown to impair lymphocyte function and inhibit immune responses, potentially leading to immunosuppression and increased susceptibility to HIV infection through altered chemokine secretion and coreceptor expression [95].

The immunosuppressive effects of opioids were initially reported in the early 1990s [120,121]. These investigations demonstrated reduced activity of NK cells, impaired macrophage phagocytosis, and decreased antibody production and T-cell function [122]. The involvement of MOR in these effects was confirmed using knockout models [123]. Generally, opioids influence NK-cell activity either through direct receptor-mediated mechanisms or indirectly via signaling pathways originating in the nervous system. However, subsequent research revealed that opioid effects on immunity are highly nuanced and environment-dependent, ranging from suppression to stimulation [105]. One such example is heroin exposure in mice, which increased pro-inflammatory cytokines (IL-2, IL-12) and nitric oxide (NO) production, while reducing anti-inflammatory cytokines (IL-4, IL-10) and accelerating skin allograft rejection [124]. Moreover, peripheral blood lymphocytes from heroin users exhibited enhanced proliferation and elevated IL-2 and IFN-γ levels, which were partially normalized by methadone maintenance therapy [125]. Methadone-treated individuals showed increased plasma levels of IL-1β, IL-6, and IL-8 [126]. Different opioids induced variable cytokine profiles; e.g., fentanyl and methadone strongly upregulated IL-4 expression, whereas morphine had a weaker effect [127]. According to reviews by Liang et al. [93] and Eisenstein [68], the effects of opioid receptor ligands on immune cells are much more complex than initially assumed. Current data from the treatment of acute and chronic pain associated with trauma, surgery, and cancer indicate that opioid receptors exert dual immunomodulatory roles influenced by opioid type, dose, duration, and physiological context, with effects spanning immunosuppression to immunostimulation [71]. Recent studies highlight the significant impact of opioids on antitumor immunity and the effectiveness of immunotherapies [128]. The authors highlighted the ability of opioids to suppress antitumor immunity by impairing CD8^+^ T-cell function and altering cytokine profiles. They also discussed how opioid use may reduce the effectiveness of immune checkpoint inhibitors and suggested that peripherally acting opioid antagonists could help mitigate these immunosuppressive effects. Furthermore, peripherally acting μ-opioid receptor antagonists (PAMORAs, represented by clinically used naloxegol, methylnaltrexone, naldemedine, and alimopan) have been proposed to address opioid-induced immunosuppression [129]. These agents block the effects of opioids outside the CNS, preserving analgesia while restoring immune function and enhancing the response to immunotherapy in preclinical models.

Specifically, opioids modulate Toll-like receptor 4 (TLR4) signaling bidirectionally by activating TLR4 in the CNS to trigger NF-κB pathways and pro-inflammatory cytokine release (TNF-α, IL-1β, IL-6), leading to neuroinflammation, while inhibiting lipopolysaccharide (LPS)-induced TLR4 signaling in peripheral immune cells, resulting in immunosuppression [130]. Both TLR4 and opioid receptors activate the MAPK pathway, contributing to neuroinflammation and pro-inflammatory effects. However, the precise mechanisms of activation can vary depending on cell type and specific opioid used, indicating complexity in these interactions [131].

Thus, this crosstalk involves complex interactions, including cytokine regulation, receptor signaling, and modulation of inflammatory responses, suggesting a fundamental biological communication mechanism between the nervous and immune systems.

As evidence of the functional linkage between the dendritic cell (DC) and opioid receptor systems reported by Li et al. [132], murine DCs displayed activation-dependent expression of functional MOR. Stimulation of DCs with the endogenous peptide endomorphin-1 (Tyr-Pro-Trp-Phe-NH_2_, EM1) inhibited cAMP formation and modulated MAPK signaling by reducing p38 activation while enhancing extracellular signal-regulated kinase (ERK). EM1 treatment significantly altered cytokine profiles, increasing IL-10 levels and decreasing IL-12 and IL-23 levels. Bidirectional interactions were also investigated in another study [133], which again explored the mechanisms by which LPS exposure influences MOR expression in immune and neuronal cells. The findings indicated that LPS modulates MOR expression through ROS-dependent signaling, highlighting the complex crosstalk between inflammatory stimuli and opioid signaling within the neuroimmune axis. Franchi et al. [107] described the immunomodulatory effects of morphine on murine macrophages, focusing on its interaction with TLR4 through MOR activation involving G(i)-protein signaling. Cuitavi et al. [134] also suggested that MORs function alongside TLRs to enhance proinflammatory mediator release during pathological conditions.

The aforementioned studies provide interesting evidence for the functional interplay between opioid receptor activation and immune signaling pathways and offer valuable insight into the immunomodulatory roles of opioids, extending beyond their classical analgesic functions to include regulation of inflammatory responses. This knowledge provides a basis for exploring how pathway-specific opioid modulation could be used in MS to attenuate neuroinflammation, support remyelination and neuroprotection, and avoid side effects inherent to opioid use.

## 6. Opioid Signaling in MS

When considering the importance of opioid signaling in MS, the following question arises: How does disease onset influence modulation of endogenous opioid levels in individuals? Two studies examined this issue in RRMS patients compared to healthy controls. Gironi et al. [135] found reduced β-endorphin levels in peripheral blood mononuclear cells (PBMCs) of untreated, clinically stable MS patients compared to healthy controls, whereas Patel et al. [136] observed significantly elevated serum OGF levels in MS patients receiving DMTs (glatiramer acetate, dimethyl fumarate, natalizumab), ranging from 193.3 to 393.4 pg/mL compared to 98.46 pg/mL in controls. Both studies indicate that opioid levels are dynamically regulated in MS. β-endorphin levels increased during clinical relapses compared to stable disease [135], while OGF levels correlated strongly with the levels of the inflammatory cytokines TNFα and IL-17A [136]. In these studies, DMTs appeared to modulate opioid production, with IFN-β significantly increasing PBMC β-endorphin levels at 1 and 3 months (*p* = 0.02 and *p* = 0.007), and glatiramer acetate uniquely elevating both OGF and serum β-endorphin. The evidence shows that opioid dysregulation in MS is complex and varies according to the immune environment rather than being uniformly increased or decreased. Untreated stable MS may be characterized by reduced cellular opioid production capacity, whereas treated patients and those with active inflammation show elevated circulating opioid levels. Thus, both studies suggest that opioid measurements may have value as biomarkers in MS. β-endorphin may reflect disease activity [135]. According to Patel et al. [136], β-endorphin levels correlated with physical health composite scores (r = 0.70), suggesting potential utility for monitoring functional status.

An interesting study demonstrated increased levels of endogenous opioids during pregnancy, whereas a decrease in these peptides after delivery was associated with an elevated risk of disease relapse [137]. Pregnancy-related remission has been associated with increased prolactin levels, a hormone that supports remyelination in animal models and correlates with improved white matter integrity. Another clinical analysis involving 40 females with RRMS assessed during both relapse and remission, plus 10 age-matched healthy controls, showed significantly higher prolactin levels during relapse than during remission and in healthy controls [138]. Notably, KOR agonists (U-69593 and nalfurafine) increase circulating prolactin levels, suggesting a possible connection between KOR activation and prolactin-mediated myelin repair, although mouse models have not consistently confirmed this effect [139]. These findings indicate that sex-related hormonal fluctuations, including pregnancy-associated increases in endogenous opioids and prolactin, postpartum declines in these factors, and sexually dimorphic responses to KOR signaling, modulate MS disease activity.

The observed changes in endogenous opioid peptide levels indicate their dynamic physiological regulation and, together with the evidence supporting their therapeutic efficacy presented below, underscore the growing interest in the development of opioid peptides as pharmacological agents. The modulatory effects of opioid signaling on immune and neuronal pathways have been explored through clinical observations, pharmacological research, studies using animal models of MS, and human studies [41].

### 6.1. Antiproliferative Effect of the OGF/OGFr Axis

As stated earlier in this work, the OGF/OGFr axis represents a novel regulatory pathway with significant implications for MS. The axis inhibits angiogenesis, thereby reducing BBB compromise and subsequent CNS inflammation. In several mouse models of experimental autoimmune encephalomyelitis (EAE; immune-driven demyelination), activation of the OGF/OGFr system suppresses T-cell, microglial, and astrocyte proliferation, which in turn reduces demyelination and clinical disease severity [6,80,88]. Campbell et al. [140] explored whether OGF could arrest disease progression. Mice that developed clinical signs of EAE after immunization with myelin oligodendrocyte glycoprotein (MOG) were treated daily with OGF (10 mg/kg) for 40 days. The results showed that OGF–OGFr signaling induced cyclin-dependent kinase inhibitors p16- and p21-mediated G_0_/G_1_ cell-cycle arrest with suppressed proliferation, alongside reduced astrocyte activation, neuronal damage, demyelination, and T-cell proliferation. OGF administration reduced behavioral scores by 45% within 6 days and maintained attenuation throughout the study. Treated mice displayed only mild symptoms compared to severe paralysis in controls. These findings indicate that OGF halts the progression of EAE, improves motor function, and normalizes pain. Another study evaluated a mouse model of relapsing-remitting (RR) EAE, induced by immunization with proteolipid protein (PLP_139–151_) [141]. Mice were treated with OGF (10 mg/kg) for 55 days. Treatment significantly reduced clinical severity (66% lower cumulative scores), prolonged remission periods, and decreased relapse frequency and duration. Neuropathological analysis revealed that receptor-mediated inhibition of DNA synthesis suppressed cell proliferation, reducing CD3^+^ T lymphocytes, Iba-1^+^ microglia/macrophages, and activated astrocytes, indicating that OGF limits CNS damage by targeting cellular proliferation. A similar study with the same mouse model of RR-EAE (immunization with PLP_139–151_) evaluated the efficiency of daily OGF administration (10 mg/kg) for 40 days [142]. OGF treatment significantly reduced clinical signs, increased remission frequency and duration, decreased relapses, and maintained mild disease severity. Neuropathology showed reduced numbers of Iba-1^+^ and CD3^+^ cells and fewer activated astrocytes, indicating inhibition of microglial/macrophage and T-cell proliferation. These findings suggest that OGF initiated after disease onset can reverse RR-EAE progression and mitigate CNS pathology.

### 6.2. KOR Targeting and Remyelination

Expanding on opioid receptor biology, the KOR has emerged as a promising therapeutic target in MS [42,143]. KOR activation may mitigate pathological changes in the EAE model through a dual mechanism: directly by promoting oligodendrocyte maturation and remyelination [143], and indirectly by modulating immune responses (reducing pathogenic T-cell cytokines) and shifting CNS-resident glial cells toward reparative phenotypes [144].

A study highlighting the therapeutic potential of KOR ligands in MS employed the selective KOR agonist MR2034 (a benzomorphan derivative) in the EAE rat model, in which myelin basic protein (MBP) was used to induce demyelination [145]. Treatment with MR2034 (at 0.2 mg/kg) significantly reduced the clinical symptoms of EAE, lowered anti-MBP antibody levels, and diminished histological changes in the brain and spinal cord. This early work established KORs as therapeutic targets, with later research confirming that KOR agonists show promise in demyelinating conditions by reducing inflammation and promoting repair. Further investigations revealed that KOR-deficient mice exhibit increased vulnerability to MS-like symptoms in the EAE model, while KOR activation alleviates symptoms and promotes oligodendrocyte-mediated remyelination [42]. Interestingly, slightly lower susceptibility was observed after the genetic deletion of DOR, whereas MOR knockout did not cause significant changes in disease progression. KOR’s protective role appears to be independent of direct immune modulation. These findings align with data showing that oligodendrocyte precursor cells (OPCs) from C57BL/6 mice express KOR and DOR, but not MOR [145]. Treatment with the KOR agonist U50488 and asimadoline significantly reduced EAE scores [42], with U50488 (1.6 mg/kg) showing optimal efficacy. Histological analysis confirmed reduced leukocyte infiltration and demyelination in the spinal cord. The therapeutic effect was absent in KOR-deficient mice, confirming receptor specificity. Importantly, KOR activation did not alter peripheral immune cell populations (CD4^+^ and CD8^+^ T cells, B cells, monocytes) or cytokine profiles (IFN-γ, IL-17A), indicating that KOR does not modulate EAE via systemic immune mechanisms. Bone marrow chimera and adoptive transfer experiments further demonstrated that KOR’s protective role is localized to CNS-resident cells. In both immune-mediated (EAE) and non-immune toxin-induced (cuprizone, a copper-chelating agent used for systemic induction of CNS changes) demyelination models, KOR activation by U50488 enhanced myelin repair, as evidenced by reduced g-ratios and increased maturation of NG2^+^ OPCs. In vitro, U50488 promoted OPC differentiation and myelination via Gαi/o signaling, L-type calcium channels, and p38 MAPK, effects abolished in KOR-deficient cultures.

In another study, Mei et al. [146] screened ~250 small molecules targeting GPCRs using a micropillar array platform (“BIMA”) to identify receptors that promote oligodendrocyte differentiation and remyelination. In purified rat OPC cultures, among all KOR agonists, treatment with U50488 increased the number of MBP-positive mature oligodendrocytes and reduced platelet-derived growth factor receptor alpha (PDGFRα) expression, indicating enhanced differentiation and suggesting that more OPCs are maturing into myelin-producing oligodendrocytes, a desirable effect in remyelination therapies for MS. In OPC-DRG cocultures, U50488 promoted the formation of myelin-like structures, suggesting increased myelination. Moreover, KOR expression was confirmed in OPCs and the corpus callosum, with immunostaining showing localization in NG2^+^ cells. Additionally, the pro-differentiation effect of U50488 was blocked by selective antagonists (nor-binaltorphimine (nor-BNI), 5′-guanidinonaltrindole (GNTI)) and absent in KOR-deficient OPCs, confirming receptor specificity. Conditional KOR deletion in OPCs (by crossing a floxed KOR mouse line with the Olig2-Cre) delayed early postnatal myelination, though recovery occurred by 6 weeks. In a lysolecithin-induced demyelination model, oral U50488 (10 mg/kg/day) enhanced remyelination in wild-type but not KOR conditional knockout mice (KOR-cKO). Human induced pluripotent stem cell (iPSC)-derived OPCs also showed increased maturation following KOR agonist treatment (1 µM for 10 days), supporting translational relevance. This study once again emphasized that KOR agonism promotes oligodendrocyte differentiation and remyelination, making it a promising target for MS therapy.

A novel series of quinoxaline-based compounds considered peripherally active, potent, and selective KOR agonists were tested for their effects on neuroinflammation and EAE [147]. The 4-N-substituted fluoroethyltriazole derivative significantly modulated KOR-dependent immune responses by decreasing IFN-γ and increasing IL-10 levels in human and mouse PBMCs, suggesting induction of tolerogenic immune pathways, and reduced EAE severity and CNS T-cell infiltration, albeit less effectively than U-50488. According to the authors, this difference could be due to the limited penetration of quinoxaline-based compounds into the CNS, suggesting that their therapeutic effect may rely more on immune modulation than on remyelination.

Another selective clinically approved KOR agonist, nalfurafine, originally developed as an analgesic but later repurposed for its antipruritic (anti-itch) effects with favorable tolerability, has shown promise in promoting remyelination and reducing neuroinflammation in EAE models of MS [148]. Nalfurafine (0.01 mg/kg) promoted recovery and remyelination in EAE, outperforming U50488, especially when administered after chronic demyelination. Its effects were KOR-dependent, as shown by nor-BNI blockade. Nalfurafine reduced CNS infiltration of CD4^+^ and CD8^+^ T cells and suppressed Th17 responses, fostering a more immunoregulatory environment. In the cuprizone model, nalfurafine enhanced remyelination even in the absence of peripheral immune involvement, affirming its direct effect on oligodendrocytes.

A synthetic analog of salvinorin A (EOM SalB), a G-protein-biased KOR agonist, showed therapeutic potential in EAE and cuprizone-induced demyelination models [149]. In EAE, EOM SalB (0.3 mg/kg) reduced disease severity, increased recovery rates, and outperformed U50488. It also decreased immune cell infiltration, particularly CD4^+^ T cells, and lowered IFN-γ and IL-17 expression in splenocytes, indicating anti-inflammatory effects. The analog enhanced CNS myelination. In the cuprizone model, EOM SalB promoted oligodendrocyte maturation, increased myelinated axon numbers, and improved myelin thickness in the corpus callosum. These effects were KOR-dependent and suggest that EOM SalB is a promising candidate for remyelinating therapies in MS and related disorders.

An interesting class of compounds considered hypothetical candidates for the treatment of MS is cyclotides, plant-derived peptides characterized by a cyclic cystine knot structure, which provides exceptional stability against heat, enzymatic degradation, and chemical breakdown [150]. These peptides are emerging as a novel class of ligands for GPCRs. The cyclotide [T20K]kalata B1, currently in clinical development for MS, was shown to bind and fully activate KOR at low micromolar concentrations. It suppressed IL-2 receptor expression, IL-2 secretion, and IL-2 gene expression in activated T cells, similar to the immunosuppressant cyclosporin A [151]. The antiproliferative effect of [T20K]kalata B1 was reversed by exogenous IL-2, indicating an IL-2-dependent mechanism. The compound also temporarily reduced IFN-γ and TNF-α production, while degranulation activity remained suppressed, suggesting interference with T-cell polyfunctionality. Orally administered [T20K]kalata B1 significantly delayed disease onset and reduced symptoms in the EAE mouse model [152]. The treatment markedly suppressed disease progression without inducing adverse effects. Notably, [T20K]kalata B1 inhibited T-cell proliferation and reduced secretion of IL-2, IFN-γ, and IL-17A in a dose-dependent manner in splenocytes from MOG-immunized mice. These findings were further supported by qPCR analysis of cytokine-related mRNA and by experiments using 2D2 MOG-specific transgenic T-cell receptor (TCR) mice, which confirmed the cyclotide’s ability to suppress inflammatory cytokine release and T-cell proliferation. Additionally, pre-treatment of C57BL/6 mice with [T20K]kB1 (10 mg/kg, i.p.) significantly delayed disease onset and reduced EAE symptoms. Imaging revealed reduced inflammation in treated mice, and splenocyte restimulation showed decreased levels of IL-2, IFN-γ, and IL-17A. Histological analysis indicated preserved myelin and minimal immune cell infiltration in the CNS. Moreover, the timing of administration influenced efficacy, because early treatment yielded the greatest reduction in inflammation and demyelination. Therapeutic administration at disease onset (clinical score 2) with a single dose showed moderate cytokine suppression, while a regimen of three doses (every third day) significantly improved clinical outcomes and reduced autoimmune cytokine levels. Histology confirmed reduced axonal damage and fewer CD3^+^ and CD4^+^ T cells in the CNS.

### 6.3. Neuroprotective and Immunomodulatory Aspects of Low Doses of Naltrexone (LDN)

Multiple studies suggest that the opiate antagonist naltrexone, which preferentially blocks opioid MOR, is relatively safe for MS patients [44,153]. In a 6-month phase 2 clinical trial conducted in 40 patients with primary progressive MS (PPMS), the initial dose was 2 mg/day, which was increased to 4 mg/day at bedtime, orally [45]. It is important to note that the proposed therapeutic approach involves the administration of low doses of naltrexone (LDN), which is much lower than the standard 50 mg daily, and has also been explored in other conditions such as autoimmune diseases (e.g., Crohn’s disease) and chronic pain (fibromyalgia) [154,155]. The proposed mechanism includes transient opioid receptor blockade that triggers a compensatory upregulation of endogenous opioids (endorphins and enkephalins). As a result, increased endogenous opioid levels may help modulate pain and improve mood. However, microglial activity modulation is also important because LDN had an antagonistic effect on Toll-like receptor 4 (TLR4), which is associated with reduced neuroinflammation. Overall, Gironi et al. [45] showed that LDN was well tolerated, with manageable adverse events and high retention. Meaningful reductions in spasticity and stability in disability progression were observed. The study also identified β-endorphin protein concentrations, mRNA levels of endogenous opioids, and allelic variants of the MOR gene (OPRM1). Above all, increased β-endorphin levels were observed. Moreover, there was no association between μ-opioid receptor genotype and therapeutic effect. Opioid receptor blockade with high-dose naltrexone (HDN, 10 mg/kg) versus intermittent blockade with low-dose naltrexone (LDN, 0.1 mg/kg) in a myelin oligodendrocyte glycoprotein (MOG)-induced EAE mouse model was investigated [156]. Daily treatment was administered throughout the disease course. HDN-treated mice showed no differences in neurological status or neuropathology compared to vehicle controls, while LDN treatment significantly reduced disease severity. All LDN-exposed animals displayed neuropathological evidence of EAE, but those without clinical symptoms had markedly lower levels of activated astrocytes and demyelination than symptomatic LDN-treated or vehicle-treated mice.

Continuing the research on OGF, Hammer et al. [157] also explored LDN in a mouse model of RR-EAE, induced by immunization with proteolipid protein 139–151 (PLP_139–151_). LDN (0.1 mg/kg, injected intraperitoneally for 40 days) significantly reduced behavioral scores and increased the length of remission and disease duration. While OGF directly and continuously activates OGFr to induce p16/p21-mediated cell-cycle arrest, LDN transiently blocks OGFr, leading to a compensatory increase in endogenous OGF–OGFr signaling that secondarily suppresses immune and glial cell proliferation, reducing CD4^+^ T-cell CNS infiltration, neuroinflammation, and demyelination in RR-EAE.

In another study, patient data were analyzed retrospectively over a follow-up period ranging from 1 to 50 months post-diagnosis [158]. Two cohorts were compared: patients receiving LDN as monotherapy (*n* = 23) and those treated with glatiramer acetate in combination with LDN (*n* = 31). The findings indicated that LDN, whether administered alone or as an adjunct to glatiramer acetate, was well tolerated and did not exacerbate disease symptoms during the observation period.

In a subsequent study [159], LDN treatment increased serum OGF levels in MS patients. Similar observations were reported in mice with EAE, in which LDN treatment normalized OGF levels. Furthermore, LDN did not affect OGF or β-endorphin levels in healthy mice, and β-endorphin remained unchanged in all groups. These findings not only underscore the importance of LDN but also suggest that OGF may serve as a selective biomarker for MS onset and progression and indicate potential therapeutic pathways for autoimmune disorders.

Administration of OGF and/or LDN, which transiently blocks OGFr and upregulates endogenous OGF, has been shown to reshape cytokine profiles by reducing IFN-γ and IL-10 levels and altering IL-6 expression [160]. Both prophylactic and therapeutic interventions targeting the OGF/OGFr axis in EAE have demonstrated reduced clinical severity and relapse frequency, lower vascular density in CNS white matter, and diminished inflammatory cytokine production.

However, Sharafaddinzadeh et al. [161] have reported findings that do not support the efficacy of LDN, indicating conflicting evidence in the literature. Their randomized placebo-controlled trial (96 participants, 17 weeks, LDN at a dose of 4.5 mg, nightly) found no meaningful differences in quality-of-life measures.

Although most evidence derives from preclinical models with limited clinical trial data, current findings strongly suggest that modulation of the endogenous opioid system may represent a promising therapeutic strategy for MS and related autoimmune disorders [41]. These three opioid-related strategies, OGF, KOR ligands, and LDN, evaluated as potential MS therapies, targeted different pathological aspects of the disease. OGF mostly demonstrated immunomodulatory activity by regulating immune cell proliferation and inflammatory responses. KOR ligands showed remyelination-promoting effects through enhanced oligodendrocyte differentiation and myelin repair. LDN, in turn, exhibited primarily neuroprotective properties by modulating endogenous opioid tone and glial activity to reduce neuroinflammation. Together, these approaches highlight how selective modulation of opioid pathways can address key therapeutic needs in MS: immune regulation, myelin repair, and neuroprotection. Table 2 summarizes preclinical and clinical studies investigating opioid system components in MS.

## 7. Pain in MS

Pain in MS is multifactorial, encompassing neuropathic, musculoskeletal, and comorbid pain syndromes, because not all pain experienced by MS patients is directly related to the disease’s pathophysiology. Approximately 27% of MS patients experience MS-related neuropathic pain [162], which results from demyelination and nerve damage. While opioids are generally reserved for severe pain conditions, their role in MS remains controversial due to concerns regarding efficacy, safety, and risk of dependency. A critical appraisal of the available data is therefore warranted to determine whether opioid-based interventions may have a place in the management of MS-related pain.

In a non-randomized, single-blind, placebo-controlled study, 14 patients with central neuropathic pain experienced pain reduction and positive opioid responsiveness following intravenous morphine administration (43 mg, 47 mg, 50 mg, and 25 mg; mean, 41 mg) [163]. Placebo and morphine produced less than 50% pain relief in most participants; only four patients were classified as opioid responders, achieving >50% pain reduction after high morphine doses (mean 41 mg) and >25% pain increase after naloxone administration. These findings indicate that central pain in MS is generally poorly responsive to opioids, requiring high doses for limited benefit, and do not support the routine use of strong opioids for MS-related pain.

Moreover, one study using Theiler’s murine encephalomyelitis virus (TMEV) infection model in mice may explain the limited effectiveness of opioid-based pain treatment in patients with MS [164]. A significant decrease in opioid receptor mRNA levels in both male and female mice at multiple time points post-infection was observed. Additionally, female mice showed reduced thermal analgesia (at day 90 post-infection) compared to male mice (at day 120 post-infection). The study revealed that downregulation of spinal opioid receptors may contribute to increased nociception in MS.

A systematic review of 15 experimental studies addressing non-spastic, non-trigeminal neuralgic pain noted that opioids were considered alongside antidepressants and anticonvulsants when evaluating efficacy and safety [165]. Moreover, the small number of trials involving MS patients with chronic pain limits the ability to make definitive treatment recommendations. No studies assessed combination pharmacotherapy.

In a retrospective cohort of 141 MS patients with varied symptoms, including pain, spasticity, and sleep disturbances, who received medical cannabis treatment [166], improvement in multiple MS-related symptoms was observed, primarily in pain relief (72% of patients), reduced spasticity (48%), and improved sleep (40%). A significant reduction in concomitant opioid use was observed, reflected by a decrease in daily morphine milligram equivalents among those prescribed opioid analgesics (*p* = 0.01). Reductions in muscle relaxant and benzodiazepine use were noted but did not reach statistical significance (*p* > 0.05). The most frequently reported adverse effect associated with medical cannabis was fatigue, occurring in 11% of patients.

A larger retrospective longitudinal cohort study of 14,974 individuals detailed the prevalence of and risk factors for chronic prescription opioid use among those with MS [167]. Over the 3-year follow-up period, chronic opioid use (≥90 days) declined. However, despite this trend, chronic prescription opioid use remains common among a substantial minority of veterans with MS and was linked to several biopsychosocial factors that are critical for understanding long-term opioid use risk.

The study by Marrie et al. [168] provides important population-based evidence that prescription opioid use is significantly more prevalent and prolonged in individuals with MS than in the general population, particularly among older patients and those with comorbid mood or anxiety disorders. Notably, the authors highlight the discrepancy between the high frequency of opioid prescribing and the limited evidence supporting long-term opioid efficacy in MS-related pain, underscoring an unmet need for safer and more mechanistically targeted therapeutic approaches.

In summary, no single study has yet examined pain improvement, opioid-related outcomes, and adverse events in an integrated manner. However, more rigorously designed and well-reported trials are needed to determine effective treatments for specific pain types experienced by people living with MS and to clarify the risks associated with long-term opioid use.

## 8. Discussion

The involvement of the endogenous opioid system in MS reflects its capacity to modulate immune responses, promote remyelination, and support neuroprotective mechanisms within the CNS. Clinical observations demonstrate that β-endorphin levels correlate with disease activity and functional status in MS, highlighting this endogenous opioid as a potential biomarker of disease burden [135]. This association is further reflected in physiological adaptations observed during pregnancy, a period associated with reduced relapse risk, followed by a postpartum decline that parallels increased disease activity [137]. Altogether, these findings provide a strong biological rationale for the hypothesis that opioid signaling contributes to MS pathophysiology by linking immune regulation, neuroprotection, and disease activity.

From a therapeutic perspective, targeting the endogenous opioid system, particularly through selective modulation of opioid receptor subtypes, represents a promising adjunctive strategy to existing DMTs. Combination approaches may enhance anti-inflammatory efficacy while simultaneously supporting remyelination and neuroprotection. However, potential challenges, including the risk of tolerance, dependence, and off-target effects, emphasize the need for receptor-specific ligands and optimized dosing regimens.

**Figure 4:**
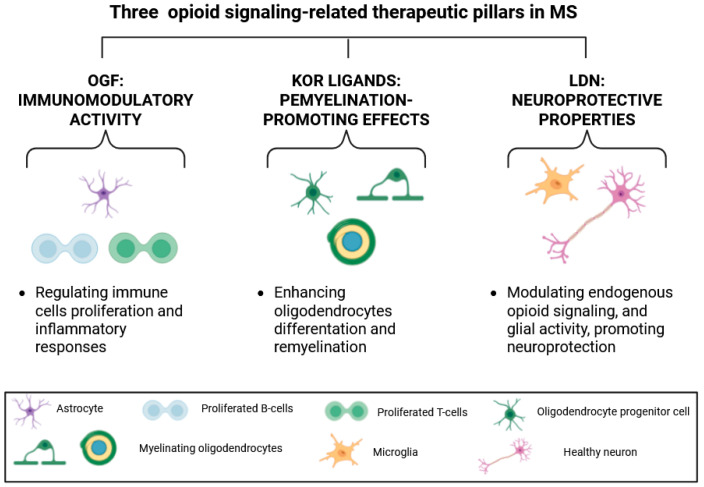
Schematic overview of three opioid signaling-related therapeutic pillars targeting key pathological mechanisms in MS. Created in https://BioRender.com.

Consistent with the multifunctional nature of opioid signaling, three opioid-related therapeutic strategies, namely OGF, KOR ligands, and LDN, address distinct yet complementary pathological dimensions of MS (Figure 4). OGF primarily exerts immunomodulatory effects by regulating immune cell proliferation and inflammatory responses. In contrast, KOR ligands promote remyelination by enhancing OPC differentiation and facilitating myelin repair. LDN predominantly demonstrates neuroprotective effects through modulation of endogenous opioid tone and glial activity, resulting in attenuation of neuroinflammation. Collectively, these approaches illustrate how selective modulation of opioid pathways can target the core features of MS pathology, including immune dysregulation, myelin loss, and neurodegeneration.

The present review uniquely integrates evidence across three interconnected therapeutic pillars—immunomodulation, remyelination, and neuroprotection—highlighting opioid signaling not merely as a symptomatic or analgesic pathway but also as a potential biomarker and DMT. This work positions the opioid system as a critical interface linking immune regulation with CNS repair mechanisms in MS.

Nevertheless, this review has limitations characteristic of its narrative nature, which preclude the generation of quantitative estimates of clinical efficacy. These constraints underscore the need for future systematic reviews, as well as well-designed longitudinal and interventional studies, to validate and expand upon the concepts presented herein and to clarify the translational potential of opioid-based therapeutic strategies in MS.

## 9. Conclusions

MS is a chronic autoimmune and neurodegenerative disease with complex pathomechanisms involving multiple cellular and molecular pathways in both the peripheral immune system and the CNS [3].

Despite significant advances in DMTs, current treatments primarily target the inflammatory component of MS and are most effective in relapsing-remitting forms. However, they offer limited efficacy in halting neurodegeneration or promoting remyelination, particularly in progressive MS subtypes. Therefore, identifying new treatments with more consistent therapeutic effects and fewer adverse effects would be highly valuable. Considerable interest has focused on strategies that, e.g., enhance OPC differentiation, while also modulating immune responses without causing harmful immunosuppression [169].

Notably, the three therapeutic pillars—immunomodulation, remyelination, and neuroprotection—may collectively represent targets of the opioid system. Despite valid safety concerns, the mechanistic diversity of endogenous opioid signaling suggests that this system may offer therapeutic benefits beyond well-established applications.

The opioid system regulates pain, stress responses, mood, and immune function, and growing evidence indicates its involvement in MS pathogenesis [41].

Accumulating scientific evidence shows that targeting the endogenous opioid system represents a promising therapeutic direction in MS. Because opioid receptors are expressed on immune cells, their activation can modulate inflammatory pathways, while opioid signaling may also promote remyelination and neuronal resilience.

An important avenue for future research is the receptor-specific modulation of the opioid system as an adjunct to existing DMTs. Current evidence most strongly supports intensive exploration and future clinical translation of KOR-dependent and other remyelination-focused strategies, especially for progressive MS, in which current DMTs remain insufficient. Immunomodulatory and neuroprotective effects mediated by OGF-related pathways and LDN are likely to remain complementary approaches and require further elucidation. However, the risks of tolerance and addiction remain important challenges that must also be addressed. Selective targeting of opioid receptor subtypes is essential to maximize therapeutic benefits while minimizing adverse effects.

## Figures and Tables

**Table 1:** Overview of the main molecular, cellular, tissue-level, and pathomorphological changes in MS.

Level	Key Changes
Molecular	Pro-inflammatory cytokine dominance: ↑ TNF-α, IFN-γ, IL-17, IL-22. Reduced regulatory cytokines: ↓ IL-10, TGF-β. Blood–brain barrier disruption. Increased adhesion molecules.
Cellular	Expansion of autoreactive Th1 and Th17 T cells. Intrathecal B-cell activation. Microglial and macrophage activation → cytokine secretion, oxidative stress. Oligodendrocyte apoptosis and impaired remyelination. Reactive astrocytes (astrogliosis) contribute to inflammation and scar formation.
Tissue	Multifocal demyelination in white and gray matter. Early axonal transection leading to progressive neurodegeneration. Perivascular inflammatory infiltrates. Diffuse microglial activation beyond visible plaques. Meningeal inflammation, especially in progressive MS. Partial remyelination forming shadow plaques.
Pathomorphological lesions	Active plaques: dense inflammation, ongoing demyelination, and myelin-laden macrophages. Chronic active (smoldering) plaques: inactive center surrounded by a rim of activated microglia. Inactive plaques: demyelinated, hypocellular areas with marked gliosis and axonal loss. Remyelinated (shadow) plaques: thin, incomplete myelin sheaths indicating partial repair.

**Table 2:** Preclinical and clinical studies investigating opioid system components in MS.

Study Design/Model	Opioid System Component	Core Biological Effect	Functional Outcomes	Ref.
Primary cultures of mouse cerebral astrocytes and an animal model for experimental autoimmune encephalomyelitis (EAE)	Opioid growth factor (OGF)- opioid growth factor receptor (OGFr) axis	OGF-OGFr signaling: ↑ p16 and p21; G_0_/G_1_ cell-cycle arrest; Inhibition of DNA synthesis; ↓ T-cell, microglia, astrocytes proliferation; ↓ neuroinflammation and demyelination.	OGF halted the progression of EAE, improves motor function, and normalizes pain	[140]
Animal model for relapse-remitting EAE (RR-EAE)	OGF-OGFr axis	OGF-OGFr signaling: Inhibition of DNA synthesis; ↓ T-cell, microglia, astrocytes proliferation; ↓ neuroinflammation and demyelination.	OGF caused fewer relapses, prolongs remission, and limits demyelination in a RR-EAE model.	[141]
Animal model for RR-EAE	OGF-OGFr axis	OGF-OGFr signaling: inhibition of DNA synthesis; G_0_/G_1_ cell-cycle arrest; ↓ T-cell, microglia, astrocytes proliferation; ↓ neuroinflammation and demyelination.	OGF improved disease control and longer remission	[142]
Animal model for RR-EAE	OGF-OGFr axis and LDN	OGF-OGFr signaling (direct) and LDN (transient blockade): ↑ p16 and p21 G_0_/G_1_ cell-cycle arrest inhibition of DNA synthesis ↓ T-cell proliferation.	OGF-OGFr signaling and LDN attenuated RR-EAE progression	[157]
Animal model for EAE and MS patients	OGF-OGFr axis and LDN	OGF-OGFr signaling (direct) and LDN (transient blockade): modulation of cytokine expression (↓ IFN-γ, altered IL-6, ↓ IL-10).	OGF-OGFr signaling and LDN attenuated autoimmune inflammation in EAE and MS without inducing global immunosuppression	[160]
Animal model for EAE	Kappa opioid receptor (KOR)	KOR agonist MR2034: neuroimmune modulation ↓ anti-myelin immune response ↓ CNS inflammation and demyelination.	MR2034 mitigated EAE severity	[145]
Animal model for EAE and in vitro oligodendrocytes model	KOR	KOR agonist U50488: Gα_i_/_o_—mediated signaling ↑ oligodendrocyte precursor cells (OPC) differentiation ↑ mature oligodendrocytes ↑ remyelination.	KOR activation reduced EAE severity, without significantly altering immune cell responses	[42]
In vitro models: OPC, oligodendrocyte–neuron co-cultures (OPC-DRG), human induced pluripotent stem cell–derived OPCs (hiPSC-OPCs) and in vivo focal demyelination model	KOR	KOR agonist U50488: ↑ OPC differentiation ↑ mature oligodendrocytes ↑ myelin formation and remyelination.	U50488 enhanced myelin repair in vitro and in vivo	[146]
Animal model for EAE	KOR	Quinoxaline-based KOR agonist: Gα_i_/_o_- mediated signaling ↓ T-cell activation ↓ antigen presenting cell (APC)-driven restimulation ↓ autoimmune neuroinflammation.	Lead quinoxaline-based KOR agonists delayed EAE onset and reduced its severity	[147]
Animal model for EAE and cuprizone-induced demyelination	KOR	Nalfurafine: Gα_i_/_o_—mediated signaling ↓ Th17 responses ↓ CD4^+^/CD8^+^ CNS infiltration ↑ OPC differentiation ↑ remyelination.	Nalfurafine reduced EAE severity and facilitated recovery in both immune-mediated and toxin models	[148]
Animal model for EAE and cuprizone-induced demyalination	KOR	EOM SalB: ↑ OPC differentiation ↑ mature oligodendrocytes ↑ myelin formation and remyelination ↓ immune cell infiltration into the CNS.	EOM SalB reduced EAE severity and facilitated recovery in both immune-mediated and toxin models	[149]
In vitro HEK293 cells, an animal model for EAE	KOR	[T20K]kalata B1: ↓ T-cell proliferation acts via an IL-2–dependent mechanism ↓ pro-inflammatory cytokines, particularly IL-2.	[T20K]kalata B1 demonstrated oral efficacy in EAE without toxicity; delayed and attenuated EAE severity	[150,152]
In vivo murine models of demyelination	KOR in glial cells	Nalfurafine: ↓ pro-inflammatory glial activation ↓ production of inflammatory mediators ↓ immune cell infiltration into the CNS ↑ remyelination.	Nalfurafine promoted a neuroprotective environment in demyelinating disease	[144]
Phase II multicenter pilot trial; patients with primary progressive MS (PPMS)	LDN	LDN: transient opioid receptor blockade ↑ endogenous opioids (OGF) ↑ opioid-mediated regulatory pathways ↓ neuroinflammation ↑ neuromodulation.	LDN in PPMS patients was safe and well-tolerated; it reduced spasticity, pain and slowed neurological progression	[45]
Animal model for EAE	LDN	LDN: transient opioid receptor blockade ↑ endogenous opioids (OGF) ↑ opioid-mediated regulatory pathways ↑ antiproliferative signaling via OGF–OGFr axi ↓ activated astrocytes ↓ demyelination ↓ neuronal injury	LDN reduced EAE severity	[156]
Randomized placebo-controlled trial; patients with RRMS or secondary-progressive MS (SPMS), disease duration >6 months.	LDN	LDN: transient opioid receptor blockade ↑ endogenous opioids (OGF) ↑ neuromodulation	LDN was well tolerated, with no serious adverse events reported; no significant improvement in quality of life	[161]
A retrospective clinical study (2006–2015); patients with relapsing-remitting MS (RRMS).	LDN	LDN: transient opioid receptor blockade ↑ endogenous opioids (OGF) ↑ antiproliferative signaling via OGF–OGFr axi	LDN, whether administered alone or as an adjunct to glatiramer acetate, was well tolerated and did not result in exacerbation of disease symptoms	[158]
Animal model for EAE and human MS patients (receiving Copaxone alone, LDN alone, or a combination of both)	OGF, LDN	LDN: transient opioid receptor blockade ↑ endogenous opioids (OGF) ↑ antiproliferative signaling *via* OGF–OGFr axi	OGF deficiency may serve as an early biomarker for MS and EAE; LDN specifically restored OGF levels in disease conditions, not in healthy animals, linking its mechanism to the OGF-OGFr axis	[159]

Arrows indicate direction of effect: ↑ stimulation/activation; ↓ inhibition/suppression.

## Data Availability

No new data were created or analyzed in this study. Data sharing is not applicable to this article.

## References

[1] Filippi M., Bar-Or A., Piehl F., Preziosa P., Solari A., Vukusic S., Rocca M. (2018). Multiple sclerosis. Nat. Rev. Dis. Prim..

[2] Attfield K.E., Jensen L.T., Kaufmann M., Friese M.A., Fugger L. (2022). The immunology of multiple sclerosis. Nat. Rev. Immunol..

[3] Woo M.S., Engler J.B., Friese M.A. (2024). The neuropathobiology of multiple sclerosis. Nat. Rev. Neurosci..

[4] Hittle M., Culpepper W.J., Langer-Gould A., Marrie R.A., Cutter G.R., Kaye W.E., Wagner L., Topol B., LaRocca N.G., Nelson L.M. (2023). Population-Based Estimates for the Prevalence of Multiple Sclerosis in the United States by Race, Ethnicity, Age, Sex, and Geographic Region. JAMA Neurol..

[5] Banci L., Bertini I., Boca M., Girotto S., Martinelli M., Valentine J.S., Vieru M. (2008). SOD1 and amyotrophic lateral sclerosis: Mutations and oligomerization. PLoS ONE.

[6] Zagon I.S., McLaughlin P.J. (2014). Endogenous Opioids and the Treatment of Multiple Sclerosis. J. Neurol. Neurophysiol..

[7] Murgia F., Giagnoni F., Lorefice L., Caria P., Dettori T., D’Alterio M.N., Angioni S., Hendren A.J., Caboni P., Pibiri M. (2022). Sex Hormones as Key Modulators of the Immune Response in Multiple Sclerosis: A Review. Biomedicines.

[8] Sabel C.E., Pearson J.F., Mason D.F., Willoughby E., Abernethy D.A., Taylor B.V. (2021). The latitude gradient for multiple sclerosis prevalence is established in the early life course. Brain.

[9] Balcerac A., Louapre C. (2022). Genetics and familial distribution of multiple sclerosis: A review. Rev. Neurol..

[10] Alfredsson L., Olsson T. (2019). Lifestyle and Environmental Factors in Multiple Sclerosis. Cold Spring Harb. Perspect. Med..

[11] Ehtesham N., Rafie M.Z., Mosallaei M. (2021). The global prevalence of familial multiple sclerosis: An updated systematic review and meta-analysis. BMC Neurol..

[12] Weber F., Fontaine B., Cournu-Rebeix I., Kroner A., Knop M., Lutz S., Müller-Sarnowski F., Uhr M., Bettecken T., Kohli M. (2008). IL2RA and IL7RA genes confer susceptibility for multiple sclerosis in two independent European populations. Genes Immun..

[13] International Multiple Sclerosis Genetics Consortium, MultipleMS Consortium (2023). Locus for severity implicates CNS resilience in progression of multiple sclerosis. Nature.

[14] Hedström A.K., Olsson T., Kockum I., Hillert J., Alfredsson L. (2020). Low sun exposure increases multiple sclerosis risk both directly and indirectly. J. Neurol..

[15] Nguyen H.D., Kim D., Kim Y.-H., Flemington E., Giovannoni G., Park C.-G., Kim W.-K. (2025). Epstein–Barr virus and multiple sclerosis: Lesson learned to develop better nonhuman primate models. Exp. Mol. Med..

[16] Wang J., Qiu Y., Marti Z., Li F., Wacker M., Oldrati P., Mühlenbruch L., Jin L., Zhang H., Xu W. (2026). EBV infection and HLA-DR15 jointly drive multiple sclerosis by myelin peptide presentation. Cell.

[17] Sintzel M.B., Rametta M., Reder A.T. (2018). Vitamin D and Multiple Sclerosis: A Comprehensive Review. Neurol. Ther..

[18] Abbasi H., Khoshdooz S., Alem E., Bakhshimoghaddam F., Doaei S., Goodarzi M.O. (2024). Vitamin D in Multiple Sclerosis: A Comprehensive Umbrella Review. J. Nutr..

[19] Wingerchuk D.M. (2012). Smoking: Effects on multiple sclerosis susceptibility and disease progression. Ther. Adv. Neurol. Disord..

[20] Mohammadi M., Mohammadi A., Habibzadeh A., Korkorian R., Mohamadi M., Shaygannejad V., Zabeti A., Mirmosayyeb O. (2024). Abnormal body mass index is associated with risk of multiple sclerosis: A systematic review and meta-analysis. Obes. Res. Clin. Pract..

[21] Hagman E., Putri R.R., Danielsson P., Marcus C. (2025). Pediatric obesity and the risk of multiple sclerosis: A nationwide prospective cohort study. Int. J. Obes..

[22] Atabilen B., Akdevelioğlu Y., Acar Özen P., Tuncer A. (2024). Examining dietary habits in the context of multiple sclerosis: A comprehensive investigative approach. Mult. Scler. Relat. Disord..

[23] Pivovarova-Ramich O., Zimmermann H.G., Paul F. (2023). Multiple sclerosis and circadian rhythms: Can diet act as a treatment?. Acta Physiol..

[24] Gerhardsson L., Hou L., Pettersson K. (2021). Work-related exposure to organic solvents and the risk for multiple sclerosis-a systematic review. Int. Arch. Occup. Environ. Health.

[25] Altieri C., Speranza B., Corbo M.R., Sinigaglia M., Bevilacqua A. (2023). Gut-Microbiota, and Multiple Sclerosis: Background, Evidence, and Perspectives. Nutrients.

[26] He R., Du Y., Wang C. (2022). Epstein–Barr virus infection: The leading cause of multiple sclerosis. Signal Transduct. Target. Ther..

[27] Chen J.J., Carletti F., Young V., Mckean D., Quaghebeur G. (2016). MRI differential diagnosis of suspected multiple sclerosis. Clin. Radiol..

[28] Wildner P., Stasiołek M., Matysiak M. (2020). Differential diagnosis of multiple sclerosis and other inflammatory CNS diseases. Mult. Scler. Relat. Disord..

[29] Yamout B., Al-Jumah M., Sahraian M.A., Almalik Y., Khaburi J.A., Shalaby N., Aljarallah S., Bohlega S., Dahdaleh M., Almahdawi A. (2024). Consensus recommendations for diagnosis and treatment of Multiple Sclerosis: 2023 revision of the MENACTRIMS guidelines. Mult. Scler. Relat. Disord..

[30] Kułakowska A., Mirowska-Guzel D., Kalinowska A., Bartosik-Psujek H., Brola W., Stasiolek M., Głąbiński A., Losy J., Potemkowski A., Rejdak K. (2024). Disease-modifying therapy in multiple sclerosis: Recommendations of Multiple Sclerosis and Neuroimmunology Section of Polish Neurological Society. Neurol. Neurochir. Pol..

[31] Rościszewska-Żukowska I., Kułakowska A., Kalinowska A., Bartosik-Psujek H., Mirowska-Guzel D., Stasiołek M., Zakrzewska-Pniewska B., Brola W., Wawrzyniak S., Gołębiowski M. (2025). Recommendations of Multiple Sclerosis and Neuroimmunology Section of Polish Neurological Society and Immuno-oncology Section of Polish Society of Oncology on oncological risk in patients with multiple sclerosis undergoing immunomodulatory therapy. Neurol. Neurochir. Pol..

[32] Montalban X., Lebrun-Frénay C., Oh J., Arrambide G., Moccia M., Pia Amato M., Amezcua L., Banwell B., Bar-Or A., Barkhof F. (2025). Diagnosis of multiple sclerosis: 2024 revisions of the McDonald criteria. Lancet Neurol..

[33] Abdelhak A., Bachhuber F., Ning K., Benkert P., John Boscardin W., Maleska Maceski A., Schaedelin S., Achtnichts L., Finkener S., Lalive P.H. (2025). Blood biomarkers for predicting disability worsening in progressive multiple sclerosis: A multinational, individual participant-level analysis. J. Neurol. Neurosurg. Psychiatry.

[34] Lin X., Tong J., Wu W., Pan X. (2025). Clinical applications and diagnostic research of GFAP and NfL in MS and NMOSD: A meta-analysis. BMC Immunol..

[35] Jatczak-Pawlik I., Jurewicz A., Domowicz M., Ewiak-Paszyńska A., Stasiołek M. (2024). CHI3L1 in Multiple Sclerosis-From Bench to Clinic. Cells.

[36] Banwell B., Bennett J.L., Marignier R., Kim H.J., Brilot F., Flanagan E.P., Ramanathan S., Waters P., Tenembaum S., Graves J.S. (2023). Diagnosis of myelin oligodendrocyte glycoprotein antibody-associated disease: International MOGAD Panel proposed criteria. Lancet Neurol..

[37] Zakrzewska-Pniewska B., Bartosik-Psujek H., Brola W., Gołębiowski M., Kalinowska A., Kułakowska A., Mirowska-Guzel D., Nojszewska M., Podlecka-Piętowska A., Stasiołek M. (2025). Update on diagnosis and treatment of neuromyelitis optica spectrum disorders (NMOSD)—Recommendations of Section of Multiple Sclerosis and Neuroimmunology of Polish Neurological Society. Neurol. Neurochir. Pol..

[38] Norborg H., Aarseth J.H., Mannseth J., Henriksen H.N., Grytten N., Myhr K.M., Wergeland S. (2025). Effect of early highly effective treatment compared to an escalating treatment strategy in multiple sclerosis. Mult. Scler. Relat. Disord..

[39] Leung M.W.Y., Garde E.M.W.V., Uitdehaag B.M.J., Klungel O.H., Bazelier M.T. (2025). The relative risk of infection in people with multiple sclerosis using disease-modifying treatment: A systematic review of observational studies. Neurol. Sci..

[40] Giannopapas V., Smyrni V., Kitsos D.K., Stefanou M.I., Theodorou A., Tzartos J.S., Tsivgoulis G., Giannopoulos S. (2025). Cancer in multiple sclerosis patients following prolonged exposure to disease-modifying therapies (DMTs): A systematic review and meta-analysis. J. Neurol..

[41] Dworsky-Fried Z., Chadwick C.I., Kerr B.J., Taylor A.M.W. (2021). Multiple Sclerosis and the Endogenous Opioid System. Front. Neurosci..

[42] Du C., Duan Y., Wei W., Cai Y., Chai H., Lv J., Du X., Zhu J., Xie X. (2016). Kappa opioid receptor activation alleviates experimental autoimmune encephalomyelitis and promotes oligodendrocyte-mediated remyelination. Nat. Commun..

[43] Valentino R.J., Volkow N.D. (2018). Untangling the complexity of opioid receptor function. Neuropsychopharmacology.

[44] Agrawal Y.P. (2005). Low dose naltrexone therapy in multiple sclerosis. Med. Hypotheses.

[45] Gironi M., Martinelli-Boneschi F., Sacerdote P., Solaro C., Zaffaroni M., Cavarretta R., Moiola L., Bucello S., Radaelli M., Pilato V. (2008). A pilot trial of low-dose naltrexone in primary progressive multiple sclerosis. Mult. Scler. J..

[46] Eva L., Pleș H., Covache-Busuioc R.-A., Glavan L.A., Bratu B.-G., Bordeianu A., Dumitrascu D.-I., Corlatescu A.D., Ciurea A.V. (2023). A Comprehensive Review on Neuroimmunology: Insights from Multiple Sclerosis to Future Therapeutic Developments. Biomedicines.

[47] de Sèze J., Maillart E., Gueguen A., Laplaud D.A., Michel L., Thouvenot E., Zephir H., Zimmer L., Biotti D., Liblau R. (2023). Anti-CD20 therapies in multiple sclerosis: From pathology to the clinic. Front. Immunol..

[48] Correale J., Marrodan M., Ysrraelit M.C. (2019). Mechanisms of Neurodegeneration and Axonal Dysfunction in Progressive Multiple Sclerosis. Biomedicines.

[49] Multz R.A., Jamshidi P., Ahrendsen J.A. (2025). Multiple sclerosis: A practical review for pathologists. J. Pathol. Transl. Med..

[50] Coclitu C.I., Constantinescu C.S., Tanasescu R. (2025). Neuroprotective strategies in multiple sclerosis: A status update and emerging paradigms. Expert Rev. Neurother..

[51] Sun X., Zhang F., Wang L., Lee G., Yang S., Zhou D., Chang B., Hu B., Zhou Y. (2025). Immunological microenvironment and targeted therapeutics in multiple sclerosis: New insights in crosstalk between immune niches and CNS. Front. Immunol..

[52] Zuroff L., Farkhondeh V., Bove R., Green A.J. (2025). The Road to Remyelination in Multiple Sclerosis: Breakthroughs, Challenges, and Considerations for Future Trial Design. Drugs.

[53] Filippi M., Preziosa P., Rocca M.A. (2025). Randomized controlled trials for multiple sclerosis: Integrating pathology-driven outcomes to capture therapeutic efficacy. J. Neurol..

[54] Rullo L., Morosini C., Lacorte A., Cristani M., Coluzzi F., Candeletti S., Romualdi P. (2024). Opioid system and related ligands: From the past to future perspectives. J. Anesth. Analg. Crit. Care.

[55] Stein C. (2016). Opioid Receptors. Annu. Rev. Med..

[56] Pasternak G.W., Abbadie C., Gebhart G.F., Schmidt R.F. (2013). Opioid Receptor Localization. Encyclopedia of Pain.

[57] Reeves K.C., Shah N., Muñoz B., Atwood B.K. (2022). Opioid Receptor-Mediated Regulation of Neurotransmission in the Brain. Front. Mol. Neurosci..

[58] Coutens B., Ingram S.L. (2023). Key differences in regulation of opioid receptors localized to presynaptic terminals compared to somas: Relevance for novel therapeutics. Neuropharmacology.

[59] Erbs E., Faget L., Scherrer G., Matifas A., Filliol D., Vonesch J.-L., Koch M., Kessler P., Hentsch D., Birling M.-C. (2015). A mu–delta opioid receptor brain atlas reveals neuronal co-occurrence in subcortical networks. Brain Struct. Funct..

[60] Cole R.H., Allichon M.C., Joffe M.E. (2025). Opioid Receptors Modulate Inhibition within the Prefrontal Cortex through Dissociable Cellular and Molecular Mechanisms. J. Neurosci..

[61] Machelska H., Celik M.Ö. (2020). Opioid Receptors in Immune and Glial Cells—Implications for Pain Control. Front. Immunol..

[62] Zhang H., Largent-Milnes T.M., Vanderah T.W. (2020). Glial neuroimmune signaling in opioid reward. Brain Res. Bull..

[63] Green J.M., Sundman M.H., Chou Y.H. (2022). Opioid-induced microglia reactivity modulates opioid reward, analgesia, and behavior. Neurosci. Biobehav. Rev..

[64] Wei J., Lambert T.Y., Valada A., Patel N., Walker K., Lenders J., Schmidt C.J., Iskhakova M., Alazizi A., Mair-Meijers H. (2023). Single nucleus transcriptomics of ventral midbrain identifies glial activation associated with chronic opioid use disorder. Nat. Commun..

[65] Ray S., Datta S., Saha A., Sil S. (2025). Astrocytes and Astrocyte-Derived Extracellular Conduits in Opiate-Mediated Neurological Disorders. Cells.

[66] Wu W., Li Q., Yang F. (2025). The actions of morphine on microglia and the underlying effects on associated adverse effects. Psychopharmacology.

[67] Gonzalez-Espinosa C., Madera-Salcedo I.K., Molina-Martínez L.M., Martínez-Cuevas F.L., Cruz S.L. (2002). Opioids and the Immune System. Opioids.

[68] Eisenstein T.K. (2019). The Role of Opioid Receptors in Immune System Function. Front. Immunol..

[69] Gavériaux-Ruff C., Simonin F., Peluso J., Befort K., Kieffer B. (1994). Expression of opioid receptors mRNAs in immune cells. Regul. Pept..

[70] Chuang T.K., Killam K.F., Chuang L.F., Kung H.F., Sheng W.S., Chao C.C., Yu L., Chuang R.Y. (1995). Mu opioid receptor gene expression in immune cells. Biochem. Biophys. Res. Commun..

[71] Brejchova J., Holan V., Svoboda P. (2020). Expression of Opioid Receptors in Cells of the Immune System. Int. J. Mol. Sci..

[72] Plein L.M., Rittner H.L. (2018). Opioids and the immune system—Friend or foe. Br. J. Pharmacol..

[73] Bidlack J.M. (2000). Detection and function of opioid receptors on cells from the immune system. Clin. Diagn. Lab. Immunol..

[74] McCarthy L., Szabo I., Nitsche J.F., Pintar J.E., Rogers T.J. (2001). Expression of functional mu-opioid receptors during T cell development. J. Neuroimmunol..

[75] Mazahery C., Valadkhan S., Levine A.D. (2019). Signaling from individual Opioid Receptors differentially modulates the functional and phenotypic potential of resting and activated human CD8+ T cells. J. Immunol..

[76] Maduna T., Audouard E., Dembélé D., Mouzaoui N., Reiss D., Massotte D., Gaveriaux-Ruff C. (2019). Microglia Express Mu Opioid Receptor: Insights From Transcriptomics and Fluorescent Reporter Mice. Front. Psychiatry.

[77] Zhang L., Belkowski J.S., Briscoe T., Rogers T.J. (2012). Regulation of mu opioid receptor expression in developing T cells. J. Neuroimmune Pharmacol..

[78] Nakamoto K., Tokuyama S. (2023). Stress-Induced Changes in the Endogenous Opioid System Cause Dysfunction of Pain and Emotion Regulation. Int. J. Mol. Sci..

[79] Swingler M., Donadoni M., Unterwald E.M., Maggirwar S.B., Sariyer I.K. (2025). Molecular and cellular basis of mu-opioid receptor signaling: Mechanisms underlying tolerance and dependence development. Front. Neurosci..

[80] Zagon I.S., McLaughlin P.J. (2024). Biology of the Opioid Growth Factor—Opioid Growth Factor Receptor Axis: Bench to Bedside and Back. Med. Res. Arch..

[81] Przewlocki R., Pfaff D.W., Volkow N.D., Rubenstein J. (2022). Opioid Peptides and Their Receptors. Neuroscience in the 21st Century.

[82] Holdridge S., Kabli N., Cahill C., Schmidt R., Willis W. (2007). Opioid Peptide Co-Localization and Release. Encyclopedia of Pain.

[83] Xu Y., Chen R., Zhi F., Sheng S., Khiati L., Yang Y., Peng Y., Xia Y. (2023). δ-opioid Receptor, Microglia and Neuroinflammation. Aging Dis..

[84] Hakim S., Jain A., Woolf C.J. (2024). Immune drivers of pain resolution and protection. Nat. Immunol..

[85] Coffey K.R., Neumaier J.F. (2024). A unique role for cAMP signaling in microglia during opioid tolerance and withdrawal. Neuropsychopharmacology.

[86] Huang H., Liu B., Qu N., Zhang S., Bai X., Handley M., Shan F. (2021). Research progress of opioid growth factor in immune-related diseases and cancer diseases. Int. Immunopharmacol..

[87] Hankins G.R., Harris R.T. (2024). The Opioid Growth Factor in Growth Regulation and Immune Responses in Cancer. Adv. Neurobiol..

[88] Zagon I.S., Verderame M.F., McLaughlin P.J. (2002). The biology of the opioid growth factor receptor (OGFr). Brain Res. Brain Res. Rev..

[89] Rogers T.J., Liu-Chen L.Y., Inan S. (2022). Kappa Opioid Receptor Expression and Function in Cells of the Immune System. The Kappa Opioid Receptor.

[90] Börner C., Kraus J., Bedini A., Schraven B., Höllt V. (2008). T-Cell Receptor/CD28-Mediated Activation of Human T Lymphocytes Induces Expression of Functional μ-Opioid Receptors. Mol. Pharmacol..

[91] Börner C., Warnick B., Smida M., Hartig R., Lindquist J.A., Schraven B., Höllt V., Kraus J. (2009). Mechanisms of opioid-mediated inhibition of human T cell receptor signaling. J. Immunol..

[92] Suno-Ikeda C., Nishikawa R., Suzuki R., Yokoi S., Iwata S., Takai T., Ogura T., Hirose M., Tokuda A., Katamoto R. (2025). Structural and dynamic insights into the biased signaling mechanism of the human kappa opioid receptor. Nat. Commun..

[93] Liang X., Liu R., Chen C., Ji F., Li T. (2016). Opioid System Modulates the Immune Function: A Review. Transl. Perioper. Pain Med..

[94] Carr D.J. (1991). The role of endogenous opioids and their receptors in the immune system. Proc. Soc. Exp. Biol. Med..

[95] Moyano J., Aguirre L. (2019). Opioids in the immune system: From experimental studies to clinical practice. Rev. Assoc. Med. Bras..

[96] Przewłocki R., Hassan A.H., Lason W., Epplen C., Herz A., Stein C. (1992). Gene expression and localization of opioid peptides in immune cells of inflamed tissue: Functional role in antinociception. Neuroscience.

[97] Mousa S.A., Shakibaei M., Sitte N., Schäfer M., Stein C. (2004). Subcellular pathways of b-Endorphin synthesis, processing, and release from immunocytes in inflammatory pain. Endocrinology.

[98] Schäfer M., Carter L., Stein C. (1994). Interleukin 1 beta and corticotropin-releasing factor inhibit pain by releasing opioids from immune cells in inflamed tissue. Proc. Natl. Acad. Sci. USA.

[99] Kapitzke D., Vetter I., Cabot P.J. (2005). Endogenous opioid analgesia in peripheral tissues and the clinical implications for pain control. Ther. Clin. Risk Manag..

[100] Cabot P.J. (2001). Immune-Derived Opioids And Peripheral Antinociception. Clin. Exp. Pharmacol. Physiol..

[101] Machelska H. (2007). Targeting of opioid-producing leukocytes for pain control. Neuropeptides.

[102] Wybran J., Appelboom T., Famaey J.P., Govaerts A. (1979). Suggestive evidence for receptors for morphine and methionine-enkephalin on normal human blood T lymphocytes. J. Immunol..

[103] Carr D.J., Bost K.L., Blalock J.E. (1988). The production of antibodies which recognize opiate receptors on murine leukocytes. Life Sci..

[104] McCarthy L., Wetzel M., Sliker J.K., Eisenstein T.K., Rogers T.J. (2001). Opioids, opioid receptors, and the immune response. Drug Alcohol Depend..

[105] Al-Hashimi M., Scott S.W., Thompson J.P., Lambert D.G. (2013). Opioids and immune modulation: More questions than answers. Br. J. Anaesth..

[106] Kraus J., Börner C., Giannini E., Hickfang K., Braun H., Mayer P., Hoehe M.R., Ambrosch A., König W., Höllt V. (2001). Regulation of μ-Opioid Receptor Gene Transcription by Interleukin-4 and Influence of an Allelic Variation Within a STAT6 Transcription Factor Binding Site. J. Biol. Chem..

[107] Franchi S., Moretti S., Castelli M., Lattuada D., Scavullo C., Panerai A.E., Sacerdote P. (2012). Mu Opioid Receptor Activation Modulates Toll Like Receptor 4 in Murine Macrophages. Brain Behav. Immun..

[108] Wang J., Charboneau R., Balasubramanian S., Barke R.A., Loh H.H., Roy S. (2002). The immunosuppressive effects of chronic morphine treatment are partially dependent on corticosterone and mediated by the μ-opioid receptor. J. Leukoc. Biol..

[109] Stein C., Hassan A.H., Przewlocki R., Gramsch C., Peter K., Herz A. (1990). Opioids from immunocytes interact with receptors on sensory nerves to inhibit nociception in inflammation. Proc. Natl. Acad. Sci. USA.

[110] Cabot P.J., Carter L., Gaiddon C., Zhang Q., Schäfer M., Loeffler J.P., Stein C. (1997). Immune Cell-derived beta-endorphin: Production, release, and control of inflammatory pain in rats. J. Clin. Investig..

[111] Labuz D., Schmidt Y., Schreiter A., Rittner H.L., Mousa S.A., Machelska H. (2009). Immune cell-derived opioids protect against neuropathic pain in mice. J. Clin. Investig..

[112] Mellon R.D., Bayer B.M. (1998). Evidence for central opioid receptors in the immunomodulatory effects of morphine: Review of potential mechanism(s) of action. J. Neuroimmunol..

[113] Vuong C., Van Uum S.H.M., O’Dell L.E., Lutfy K., Friedman T.C. (2010). The effects of opioids and opioid analogs on animal and human endocrine systems. Endocr. Rev..

[114] Alonzo N.C., Bayer B.M. (2002). Opioids, immunology, and host defenses of intravenous drug abusers. Infect. Dis. Clin..

[115] Welters I.D. (2003). Is immunomodulation by opioid drugs of clinical relevance?. Curr. Opin. Anaesthesiol..

[116] Salzet M., Vieau D., Day R. (2000). Crosstalk between nervous and immune systems through the animal kingdom: Focus on opioids. Trends Neurosci..

[117] Toloff K., Woodcock E.A. (2022). Is the Neuroimmune System a Therapeutic Target for Opioid Use Disorder? A Systematic Review. Med. Res. Arch..

[118] Azzoni L., Giron L.B., Vadrevu S., Zhao L., Lalley-Chareczko L., Hiserodt E., Fair M., Lynn K., Trooskin S., Mounzer K. (2022). Methadone use is associated with increased levels of sCD14, immune activation, and inflammation during suppressed HIV infection. J. Leukoc. Biol..

[119] Dang C.M., Nelson C.M., Feaster D.J., Kizhner A., Forrest D.W., Nakamura N., Iyer A., Ghanta P.P., Jayaweera D.T., Rodriguez A.E. (2023). Opioids exacerbate inflammation in people with well-controlled HIV. Front. Immunol..

[120] Bryant H.U., Roudebush R.E. (1990). Suppressive effects of morphine pellet implants on in vivo parameters of immune function. J. Pharmacol. Exp. Ther..

[121] Pacifici R., Minetti M., Zuccaro P., Pietraforte D. (1995). Morphine affects cytostatic activity of macrophages by the modulation of nitric oxide release. Int. J. Immunopharmacol..

[122] Sacerdote P., Manfredi B., Mantegazza P., Panerai A.E. (1997). Antinociceptive and immunosuppressive effects of opiate drugs: A structure-related activity study. Br. J. Pharmacol..

[123] Gaveriaux-Ruff C., Matthes H.W., Peluso J., Kieffer B.L. (1998). Abolition of morphine-immunosuppression in mice lacking the mu-opioid receptor gene. Proc. Natl. Acad. Sci. USA.

[124] Holan V., Zajicova A., Krulova M., Blahoutova V., Wilczek H. (2003). Augmented production of proinflammatory cytokines and accelerated allotransplantation reactions in heroin-treated mice. Clin. Exp. Immunol..

[125] Zajicova A., Wilczek H., Holan V. (2004). The alterations of immunological reactivity in heroin addicts and their normalization in patients maintained on methadone. Folia Biol..

[126] Chan Y.-Y., Yang S.-N., Lin J.-C., Chang J.-L., Lin J.-G., Lo W.-Y. (2015). Inflammatory response in heroin addicts undergoing methadone replacement therapy. Psychiatry Res..

[127] Borner C., Lanciotti S., Koch T., Hollt V., Kraus J. (2013). μ opioid receptor agonist-selective regulation of interleukin-4 in T lymphocytes. J. Neuroimmunol..

[128] Zhou Y., Li W., Chen Y., Hu X., Miao C. (2025). Research progress on the impact of opioids on the tumor immune microenvironment (Review). Mol. Clin. Oncol..

[129] Bettinger J.J., Friedman B.C. (2024). Opioids and Immunosuppression: Clinical Evidence, Mechanisms of Action, and Potential Therapies. Palliat. Med. Rep..

[130] Zhang P., Yang M., Chen C., Liu L., Wei X., Zeng S. (2020). Toll-Like Receptor 4 (TLR4)/Opioid Receptor Pathway Crosstalk and Impact on Opioid Analgesia, Immune Function, and Gastrointestinal Motility. Front. Immunol..

[131] Gabr M.M., Saeed I., Miles J.A., Ross B.P., Shaw P.N., Hollmann M.W., Parat M.O. (2021). Interaction of Opioids with TLR4-Mechanisms and Ramifications. Cancers.

[132] Li Z.H., Chu N., Shan L.D., Gong S., Yin Q.Z., Jiang X.H. (2009). Inducible expression of functional mu opioid receptors in murine dendritic cells. J. Neuroimmune Pharmacol..

[133] Langsdorf E.F., Mao X., Chang S.L. (2011). A role for reactive oxygen species in endotoxin-induced elevation of MOR expression in the nervous and immune systems. J. Neuroimmunol..

[134] Cuitavi J., Torres-Pérez J.V., Lorente J.D., Campos-Jurado Y., Andrés-Herrera P., Polache A., Agustín-Pavón C., Hipólito L. (2023). Crosstalk between Mu-Opioid receptors and neuroinflammation: Consequences for drug addiction and pain. Neurosci. Biobehav. Rev..

[135] Gironi M., Martinelli V., Brambilla E., Furlan R., Panerai A.E., Comi G., Sacerdote P. (2000). Beta-endorphin concentrations in peripheral blood mononuclear cells of patients with multiple sclerosis: Effects of treatment with interferon beta. Arch. Neurol..

[136] Patel C., Thomas G., Zomorodi N., Zagon I.S., McLaughlin P.J. (2021). β-endorphin and opioid growth factor as biomarkers of physical ability in multiple sclerosis. Mult. Scler. Relat. Disord..

[137] Costanza M., Pedotti R. (2016). Prolactin: Friend or Foe in Central Nervous System Autoimmune Inflammation?. Int. J. Mol. Sci..

[138] Alwakil H.A., Al-Malt A.M., Ragab O.A., Ghafar M.T.A., Tageldin E.A. (2020). Serum prolactin in patients with relapsing remitting multiple sclerosis. Egypt. J. Neurol. Psychiatry Neurosurg..

[139] Kopruszinski C.M., Watanabe M., Martinez A.L., de Souza L.H.M., Dodick D.W., Moutal A., Neugebauer V., Porreca F., Navratilova E. (2023). Kappa opioid receptor agonists produce sexually dimorphic and prolactin-dependent hyperalgesic priming. Pain.

[140] Campbell A.M., Zagon I.S., McLaughlin P.J. (2012). Opioid growth factor arrests the progression of clinical disease and spinal cord pathology in established experimental autoimmune encephalomyelitis. Brain Res..

[141] Hammer L.A., Zagon I.S., McLaughlin P.J. (2013). Treatment of a relapse-remitting model of multiple sclerosis with opioid growth factor. Brain Res. Bull..

[142] Hammer L.A., Zagon I.S., McLaughlin P.J. (2015). Improved clinical behavior of established relapsing-remitting experimental autoimmune encephalomyelitis following treatment with endogenous opioids: Implications for the treatment of multiple sclerosis. Brain Res. Bull..

[143] Reed B., Dutta S. (2023). The Kappa Opioid receptor: Candidate Pharmacotherapeutic target for Multiple Sclerosis. Drugs Drug Candidates.

[144] Penelgy B. (2025). Kappa Opioid Receptor Expression and Function in Glial Cells: Implications for Nalfurafine as a Therapeutic Agent in Demyelinating Diseases. Ph.D. Thesis.

[145] Radulović J., Djergović D., Miljević C., Janković B.D. (1994). kappa-Opioid receptor functions: Possible relevance to experimental allergic encephalomyelitis. Neuroimmunomodulation.

[146] Mei F., Mayoral S.R., Nobuta H., Wang F., Desponts C., Lorrain D.S., Xiao L., Green A.J., Rowitch D., Whistler J. (2016). Identification of the Kappa-Opioid Receptor as a Therapeutic Target for Oligodendrocyte Remyelination. J. Neurosci..

[147] Tangherlini G., Kalinin D.V., Schepmann D., Che T., Mykicki N., Ständer S., Loser K., Wünsch B. (2019). Development of Novel Quinoxaline-Based κ-Opioid Receptor Agonists for the Treatment of Neuroinflammation. J. Med. Chem..

[148] Denny L., Al Abadey A., Robichon K., Templeton N., Prisinzano T.E., Kivell B.M., La Flamme A.C. (2021). Nalfurafine reduces neuroinflammation and drives remyelination in models of CNS demyelinating disease. Clin. Transl. Immunol..

[149] Paton K.F., Robichon K., Templeton N., Denny L., Al Abadey A., Luo D., Prisinzano T.E., La Flamme A.C., Kivell B.M. (2021). The Salvinorin Analogue, Ethoxymethyl Ether Salvinorin B, Promotes Remyelination in Preclinical Models of Multiple Sclerosis. Front. Neurol..

[150] Muratspahić E., Tomašević N., Nasrollahi-Shirazi S., Gattringer J., Emser F.S., Freissmuth M., Gruber C.W. (2021). Plant-Derived Cyclotides Modulate κ-Opioid Receptor Signaling. J. Nat. Prod..

[151] Gründemann C., Thell K., Lengen K., Garcia-Käufer M., Huang Y.H., Huber R., Craik D.J., Schabbauer G., Gruber C.W. (2013). Cyclotides Suppress Human T-Lymphocyte Proliferation by an Interleukin 2-Dependent Mechanism. PLoS ONE.

[152] Thell K., Hellinger R., Sahin E., Michenthaler P., Gold-Binder M., Haider T., Kuttke M., Liutkevičiūtė Z., Göransson U., Gründemann C. (2016). Oral activity of a nature-derived cyclic peptide for the treatment of multiple sclerosis. Proc. Natl. Acad. Sci. USA.

[153] Patten D.K., Schultz B.G., Berlau D.J. (2018). The Safety and Efficacy of Low-Dose Naltrexone in the Management of Chronic Pain and Inflammation in Multiple Sclerosis, Fibromyalgia, Crohn’s Disease, and Other Chronic Pain Disorders. Pharmacotherapy.

[154] Dara P., Farooqui Z., Mwale F., Choe C., van Wijnen A.J., Im H.-J. (2023). Opiate Antagonists for Chronic Pain: A Review on the Benefits of Low-Dose Naltrexone in Arthritis versus Non-Arthritic Diseases. Biomedicines.

[155] Rassi-Mariani V., Barreto E.S.R., Antunes C.R., Alencar V.B., Falcão Lins-Kusterer L.E., Azi L.M.T.A., Kraychete D.C. (2024). The use of naltrexone in the treatment of chronic pain: A systematic review. Pain Manag..

[156] Zagon I.S., Rahn K.A., Turel A.P., McLaughlin P.J. (2009). Endogenous opioids regulate expression of experimental autoimmune encephalomyelitis: A new paradigm for the treatment of multiple sclerosis. Exp. Biol. Med..

[157] Hammer L.A., Waldner H., Zagon I.S., McLaughlin P.J. (2016). Opioid growth factor and low-dose naltrexone impair central nervous system infiltration by CD4 + T lymphocytes in established experimental autoimmune encephalomyelitis, a model of multiple sclerosis. Exp. Biol. Med..

[158] Ludwig M.D., Turel A.P., Zagon I.S., McLaughlin P.J. (2016). Long-term treatment with low dose naltrexone maintains stable health in patients with multiple sclerosis. Mult. Scler. J. Exp. Transl. Clin..

[159] Ludwig M.D., Zagon I.S., McLaughlin P.J. (2017). Featured Article: Serum [Met5]-enkephalin levels are reduced in multiple sclerosis and restored by low-dose naltrexone. Exp. Biol. Med..

[160] Ludwig M.D., Zagon I.S., McLaughlin P.J. (2018). Featured Article: Modulation of the OGF-OGFr pathway alters cytokine profiles in experimental autoimmune encephalomyelitis and multiple sclerosis. Exp. Biol. Med..

[161] Sharafaddinzadeh N., Moghtaderi A., Kashipazha D., Majdinasab N., Shalbafan B. (2010). The effect of low-dose naltrexone on quality of life of patients with multiple sclerosis: A randomized placebo-controlled trial. Mult. Scler. J..

[162] Rodrigues P., da Silva B., Trevisan G. (2023). A systematic review and meta-analysis of neuropathic pain in multiple sclerosis: Prevalence, clinical types, sex dimorphism, and increased depression and anxiety symptoms. Neurosci. Biobehav. Rev..

[163] Kalman S., Osterberg A., Sörensen J., Boivie J., Bertler A. (2002). Morphine responsiveness in a group of well-defined multiple sclerosis patients: A study with i.v. morphine. Eur. J. Pain.

[164] Lynch J.L., Alley J.F., Wellman L., Beitz A.J. (2008). Decreased spinal cord opioid receptor mRNA expression and antinociception in a Theiler’s murine encephalomyelitis virus model of multiple sclerosis. Brain Res..

[165] Jawahar R., Oh U., Yang S., Lapane K.L. (2013). A systematic review of pharmacological pain management in multiple sclerosis. Drugs.

[166] Rainka M.M., Aladeen T.S., Mattle A.G., Lewandowski E., Vanini D., McCormack K., Mechtler L. (2023). Multiple Sclerosis and Use of Medical Cannabis: A Retrospective Review of a Neurology Outpatient Population. Int. J. MS Care.

[167] Turner A.P., Arewasikporn A., Hawkins E.J., Suri P., Burns S.P., Leipertz S.L., Haselkorn J.K. (2023). Risk Factors for Chronic Prescription Opioid Use in Multiple Sclerosis. Arch. Phys. Med. Rehabil..

[168] Marrie R.A., Fisk J.D., Walld R., Bolton J.M., Sareen J., Patten S.B., Bernstein C.N. (2023). Prescription opioid use in multiple sclerosis. J. Neurol. Neurosurg. Psychiatry.

[169] Zveik O., Rechtman A., Ganz T., Vaknin-Dembinsky A. (2024). The interplay of inflammation and remyelination: Rethinking MS treatment with a focus on oligodendrocyte progenitor cells. Mol. Neurodegener..

